# Molecular iodine-catalyzed one-pot multicomponent synthesis of 5-amino-4-(arylselanyl)-1*H*-pyrazoles

**DOI:** 10.3762/bjoc.14.256

**Published:** 2018-11-06

**Authors:** Camila S Pires, Daniela H de Oliveira, Maria R B Pontel, Jean C Kazmierczak, Roberta Cargnelutti, Diego Alves, Raquel G Jacob, Ricardo F Schumacher

**Affiliations:** 1 LASOL - CCQFA - Universidade Federal de Pelotas - UFPel - P.O. Box 354 - 96010-900, Pelotas, RS, Brazil; 2 Departamento de Química, Universidade Federal de Santa Maria – UFSM, 97105-900, Santa Maria, RS, Brazil

**Keywords:** diaryl diselenide, diazo compound, 1*H*-pyrazole, molecular iodine, multicomponent reaction

## Abstract

A one-pot iodine-catalyzed multicomponent reaction has been developed for the selective preparation of 5-amino-4-(arylselanyl)-1*H*-pyrazoles from a diverse array of benzoylacetonitriles, arylhydrazines and diaryl diselenides. The reactions were conducted in MeCN as solvent at reflux temperature under air. The methodology presents a large functional group tolerance to electron-deficient, electron-rich, and bulky substituents and gave the expected products in good to excellent yields. The synthesized 1,3-diphenyl-4-(phenylselanyl)-1*H*-pyrazol-5-amine was submitted to an oxidative dehydrogenative coupling to produce a diazo compound confirmed by X-ray analysis.

## Introduction

**Scheme 1:**
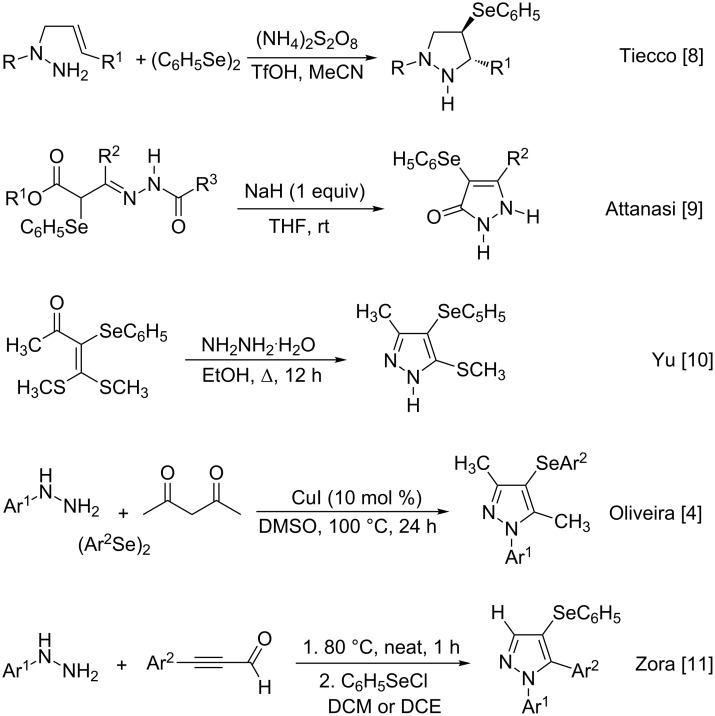
Synthesis of selanyl-pyrazoles and their derivatives previously described.

Selenium-containing compounds are of great importance in bioactive compounds, pharmaceuticals, and as chemical intermediates for organic and inorganic synthesis [1–2]. As a consequence, the development of efficient and practical methods for C–Se bond formation is still a promising field of research in organic chemistry. Despite the several number of methodologies describing the transition metal-catalyzed cross-coupling reaction of diorganyl diselenides with (hetero)aryl, alkenyl, and alkynyl halides or pseudo-halides [3], an updated protocol that describe reduced waste generation and atom-economy is desired. In this context, the multicomponent cyclization/direct selanylation is a practical and economical way to incorporate organylselanyl moieties in aryl and heteroaryl compounds avoiding the necessity of pre-functionalization, multistep synthesis and tedious work-up [4–5]. In this context, the preparation of diverse selanylated indoles and imidazopyridines, for example, is widely found in literature [6–7]. However, the preparation of 4-arylselanyl-1*H*-pyrazoles is quite scarce. Tiecco and co-workers described the preparation of phenylseleno substituted pyrazolidines by reacting allylhydrazines with phenylselanenyl sulfate generated in situ (Scheme 1) [8]. Attanasi and co-workers described the synthesis of 4-(phenylseleno)pyrazol-3-ones through α-(phenylseleno)hydrazone reagents under basic conditions [9]. In 2015, Yu and co-workers described one example for the condensation reaction of α-oxo ketene dithioacetal with hydrazine hydrate to produce the 3-methyl-5-methylsulfanyl-4-phenylselanyl-1*H*-pyrazole [10]. Our research group described the multicomponent synthesis of 3,5-dimethyl-4-arylselanyl-1*H*-pyrazoles catalyzed by copper iodide using DMSO as solvent [4]. More recently, Zora and co-workers reported the one-pot preparation of 4-phenylselanyl-1*H*-pyrazoles through reaction of α,β-alkynic hydrazones with phenylselenyl chloride [11].

In this context, pyrazoles are one of the most important N-heterocycles found in natural products including formycin, pyrazofurin and withasomnine, for example. Still, pyrazole unities are present in several commercially available drugs such as celecoxib, lonazolac, rimonabant and sildenafil [12–13]. In special, 5-amino-1*H*-pyrazoles are valuable compounds that present several biological and pharmaceutical activities including antiviral, anticancer, antituberculosis, anti-inflammatory, antifungal and antidepressive activity [14–17]. Moreover, they have been extensively used as synthons to prepare a wide variety of fused N-heterocycles of synthetic and pharmacological importance [18–20]. Thus, an effective strategy for the design and the preparation of new pharmacologically promising drugs that combine at least two bioactive moieties in one molecule avoiding residual metal in transition-metal catalysis in environmental benign conditions is desired. Recently, Sun and co-workers described their interesting results on the I_2_-catalyzed efficient synthesis of 5-amino-4-sulfanylpyrazoles using diverse β-ketonitriles, arylhydrazines and aryl sulfonyl hydrazides as sulfur source [21]. The chemistry of iodine-catalyzed transformations has emerged as a greener, efficient and economical alternative to transition metals in organic synthesis [22–24]. However, the synthesis of selenium-containing 5-aminopyrazoles under iodine-catalyzed three-component reaction was not described so far.

**Scheme 2:**
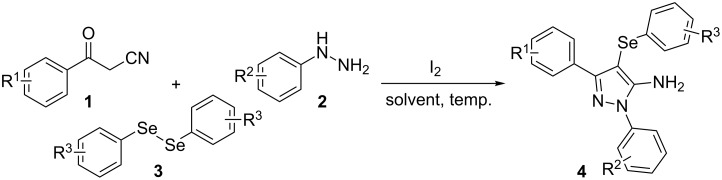
Multicomponent reaction proposed in this work.

In view of this, we present a direct and selective multicomponent strategy to synthesize 5-amino-4-(arylselanyl)-1*H*-pyrazoles **4** by reacting benzoylacetonitrile derivatives **1**, arylhydrazines **2** and diorganyl diselenides **3** (Scheme 2). The reaction is catalysed by molecular iodine in MeCN as solvent.

## Results and Discussion

Initially, to optimize the reaction conditions experiments were performed using benzoylacetonitrile (**1a**), phenylhydrazine (**2a**) and diphenyl diselenide (**3a**) as standard substrates to define the best reaction conditions. During the experiments different parameters were evaluated such as temperature, reaction time, solvent, reagent amount and heating method (conventional heating or ultrasound, Table 1).

In a first experiment, benzoylacetonitrile (**1a**, 0.5 mmol), phenylhydrazine (**2a**, 0.5 mmol) and diphenyl diselenide (**3a**, 0.5 mmol) were solubilized in MeCN (3 mL) and molecular iodine (50 mol %) was added. The mixture was stirred at reflux temperature and after 48 h under these conditions, the expected product **4a** was obtained in 71% yield (Table 1, entry 1). With these result, we attempted to investigate the parameters regarding quantities (Table 1, entries 2 and 3). Based on it, we observed that the use of a minimum amount of diphenyl diselenide (**3a**) caused a decrease in the reaction yield, giving the product **4a** in 54% (Table 1, entry 2). However, when the reaction was performed using a slight excess of **2a** (0.7 mmol) and **3a** (0.5 mmol) the reaction yield increased significantly to 96% (Table 1, entry 3). The treatment of **1a**, **2a** and **3a** in the presence of 25, 50 and 75 mol % of molecular iodine afforded the expected product **4a** in 68, 96 and 75% yield, respectively (Table 1, entries 3–5). In this sense, better results were obtained when using 50 mol % of I_2_.

In the next step, the reaction was performed in different solvents such as toluene, 1,4-dioxane and dimethylformamide, but a considerable decrease in the yield of **4a** was observed (Table 1, entries 6–8). Similarly, when the reaction was conducted at a lower temperature (Table 1, entry 9) or in a shorter reaction time (Table 1, entry 10), the product **4a** was isolated in a poorer yield. The use of ultrasonic irradiation was also tested due to the high-energy transference in the reaction during the cavitation process. Unfortunately, under this condition a low reaction yield of 42% was obtained even after 2 h of reaction (Table 1, entry 11).

**Table 1:** Optimization of the reaction conditions.^a^

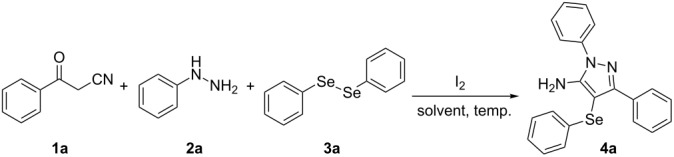							

entry	**2a** (mmol)	**3a** (mmol)	I_2_ (mol %)	solvent	temp. (°C)	time (h)	yield (%)^b^

1	0.5	0.5	50	MeCN	reflux	48	71
2	0.5	0.25	50	MeCN	reflux	48	54
3	0.7	0.5	50	MeCN	reflux	48	96
4	0.7	0.5	25	MeCN	reflux	48	68
5	0.7	0.5	75	MeCN	reflux	48	75
6	0.7	0.5	50	toluene	80	48	–
7	0.7	0.5	50	DMF	80	48	16
8	0.7	0.5	50	1,4-dioxane	80	48	55
9	0.7	0.5	50	MeCN	60	48	12
10	0.7	0.5	50	MeCN	reflux	36	68
11^c^	0.7	0.5	50	MeCN	)))	2	47

^a^Reaction was performed using **1a** (0.5 mmol), **2a** and **3a** in 3 mL of solvent followed by the addition of molecular iodine. The consumption of starting materials was followed by TLC. ^b^Yields are given for isolated products. ^c^The reaction was carried under ultrasound conditions operating at amplitude of 60% and a frequency of 20 kHz.

With the establishment of the best reaction conditions for the synthesis of **4a** described in Table 1, entry 3, we envisaged extending this methodology to diverse benzoylacetonitriles **1a**–**f**, arylhydrazines **2a**–**f** and differently substituted diaryl diselenides **3a**–**f**. In general, as shown in Table 2, most of the reactions proceeded smoothly and provided the desired products in moderate to excellent yields (see Supporting Information File 1 for full experimental data).

**Table 2:** Substrate scope for the synthesis of 5-amino-4-(arylselanyl)-1*H*-pyrazoles **4**.^a^

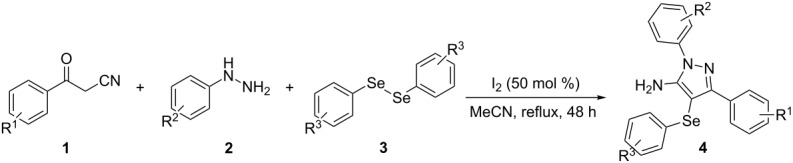				

entry	ketonitrile **1**	aryl hydrazine **2**	diaryl diselenide **3**	product **4** (yield)^b^

1	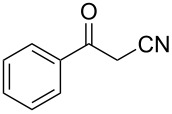 **1a**	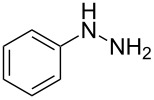 **2a**	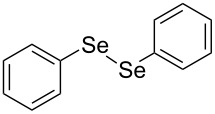 **3a**	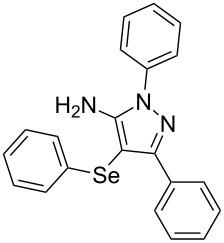 **4a** (96%)
2	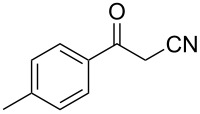 **1b**	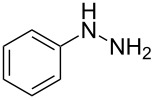 **2a**	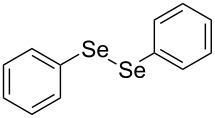 **3a**	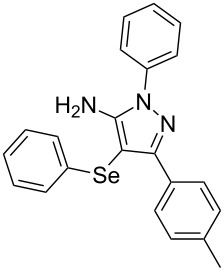 **4b** (73%)
3	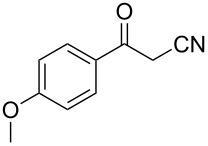 **1c**	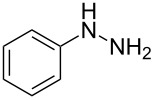 **2a**	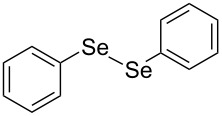 **3a**	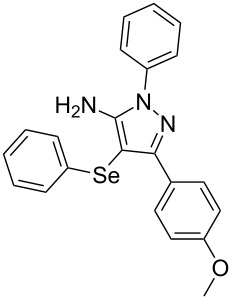 **4c** (35%)
4	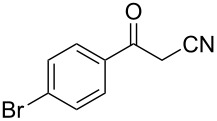 **1d**	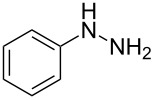 **2a**	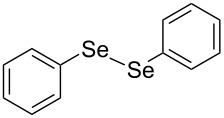 **3a**	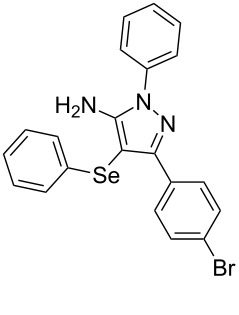 **4d** (78%)
5	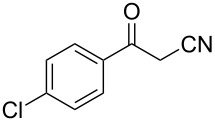 **1e**	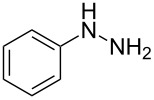 **2a**	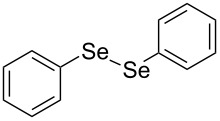 **3a**	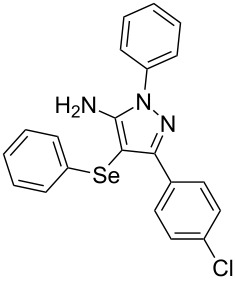 **4e** (81%)
6	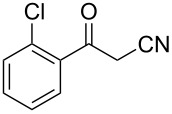 **1f**	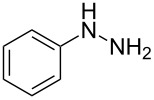 **2a**	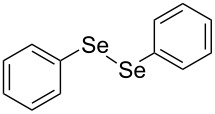 **3a**	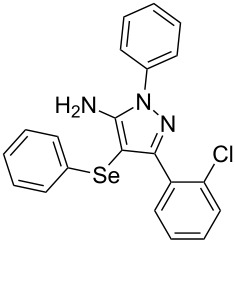 **4f** (62%)^c^
7	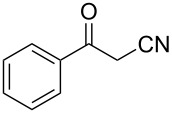 **1a**	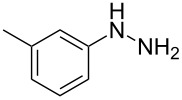 **2b**	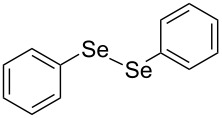 **3a**	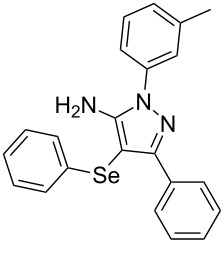 **4g** (63%)
8	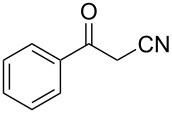 **1a**	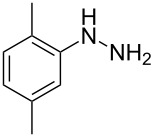 **2c**	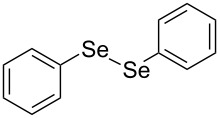 **3a**	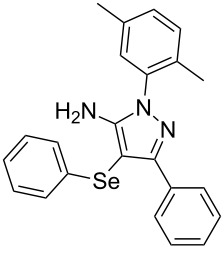 **4h** (52%)
9	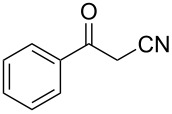 **1a**	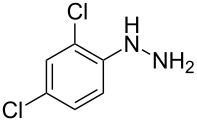 **2d**	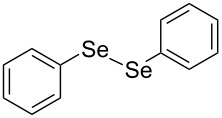 **3a**	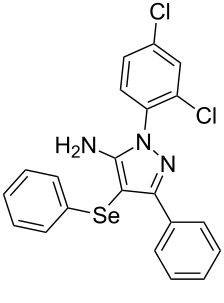 **4i** (80%)
10	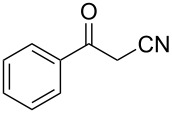 **1a**	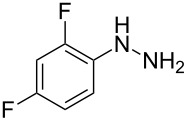 **2e**	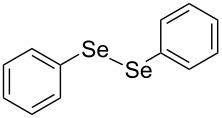 **3a**	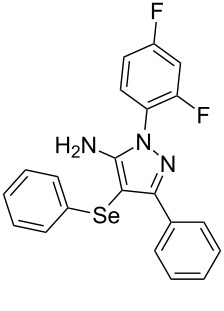 **4j** (76%)
11	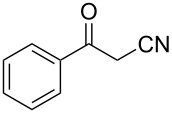 **1a**	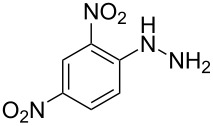 **2f**	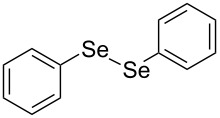 **3a**	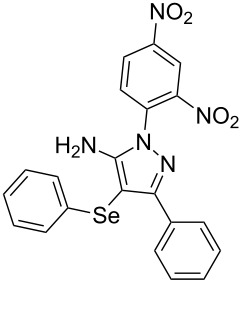 **4k** (–)^d^
12	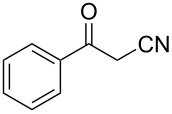 **1a**	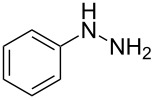 **2a**	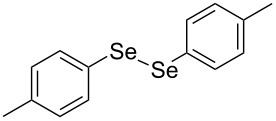 **3b**	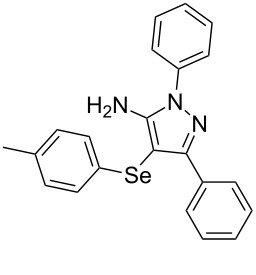 **4l** (54%)
13	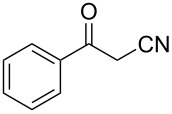 **1a**	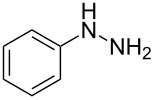 **2a**	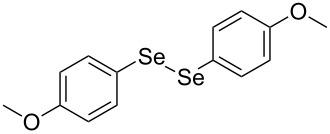 **3c**	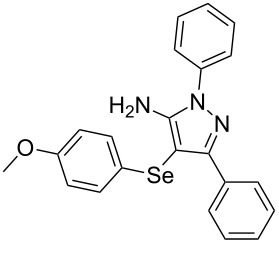 **4m** (63%)
14	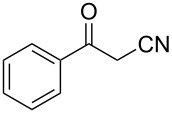 **1a**	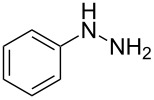 **2a**	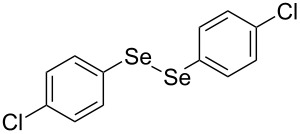 **3d**	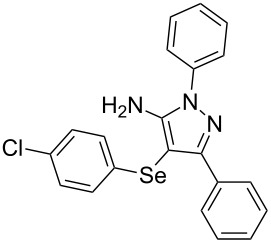 **4n** (80%)
15	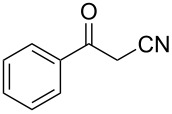 **1a**	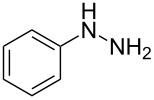 **2c**	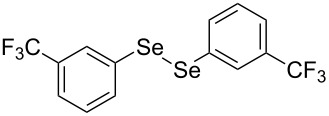 **3e**	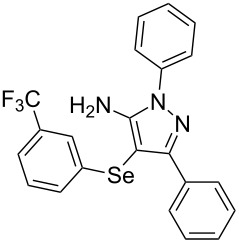 **4o** (24%)
16	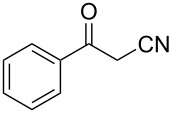 **1a**	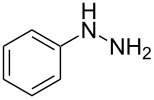 **2c**	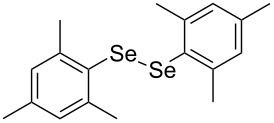 **3f**	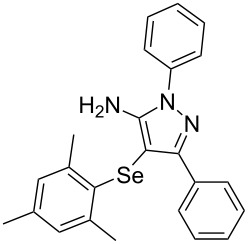 **4p** (–)^d^

^a^Reactions were performed by using **1** (0.5 mmol), **2** (0.7 mmol), **3** (0.5 mmol) and I_2_ (50 mol %) in MeCN (3 mL) at reflux temperature for 48 h in air. ^b^Yield is given for isolated products. ^c^Yield is given for a mixture of 5-amino-4-(phenylselanyl)pyrazole and 5-aminopyrazole (1:2) determined by ^1^H NMR. ^d^Product not obtained.

The substrate scope of this reaction was investigated under the optimized reaction conditions. Initially, to achieve this goal, we carried out a reaction of phenylhydrazine (**2a**) and diphenyl diselenide (**3a**) with benzoylacetonitrile **1b** containing an electron-donating group on the aromatic ring, that provided the corresponding product **4b** gently in 73% yield. When we performed the reaction by using the benzoylacetonitrile derivative **1c** substituted with a methoxy group, a lower isolated yield was observed (Table 2, entry 3). This may be associated with the high reactivity of **1c** which produced many byproducts as observed by TLC and GC–MS analysis. Benzoylacetonitriles **1d**,**e** substituted with electron-withdrawing groups such as Br and Cl were also tested and afforded the corresponding products **4d** and **4e** in good yields (Table 2, entries 4 and 5). However, when we used an *ortho*-chloro substituted benzoylacetonitrile **1f**, the corresponding product **4f** was isolated as a non-separable mixture with the non-selenylated 5-aminopyrazole (Table 2, entry 6).

Next we evaluated the reactivity of different functionalized arylhydrazines **2a**–**f** using benzoylacetonitrile **1a** and diphenyl diselenide **3a** as standard reagents (Table 2, entries 7–11). Substituted arylhydrazines were sensitive to the electronic effect of the groups directly bonded to aromatic ring. In this sense, arylhydrazines **2b** and **2c** containing EDG at the aromatic ring gave lower yields than arylhydrazines **2d** and **2e** containing EWG (Table 2, entries 7 and 8 vs 9 and 10). On the other hand, when 2,4-dinitrophenylhydrazine was used, the expected pyrazole **4k** was not detected by GC–MS analysis. (Table 2, entry 11).

In addition, different diaryl diselenides **3b**–**e** were studied to produce valuable 5-amino-4-(arylselanyl)-1*H*-pyrazoles **4l**–**o** (Table 2, entries 12–15). In this case, it is possible to see that all diaryl diselenides were efficiently coupled to 5-amino-1*H*-pyrazole to produce the corresponding products in yields ranging from 24% to 80%. However, when the bulkier 1,2-dimesityl diselenide **3f** was employed, no product was obtained (Table 2, entry 16).

**Scheme 3:**
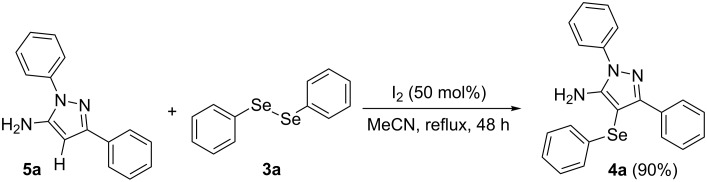
Direct selanylation reaction of 5-amino-pyrazole **5a** with diphenyl diselenide (**3a**) under the optimized conditions.

To gain insight into the mechanistic profile we also evaluated whether 5-amino-1*H*-pyrazole **5a** could produce 5-amino-4-(phenylselanyl)-1*H*-pyrazole **4a** via direct C–H bond selanylation reaction catalyzed by iodine. Thus, when **5a** reacted with diphenyl diselenide (**3a**) in the presence of 50 mol % of I_2_ in acetonitrile as solvent and under reflux, the desired product **4a** could be obtained in 90% yield as depicted in Scheme 3.

**Scheme 4:**
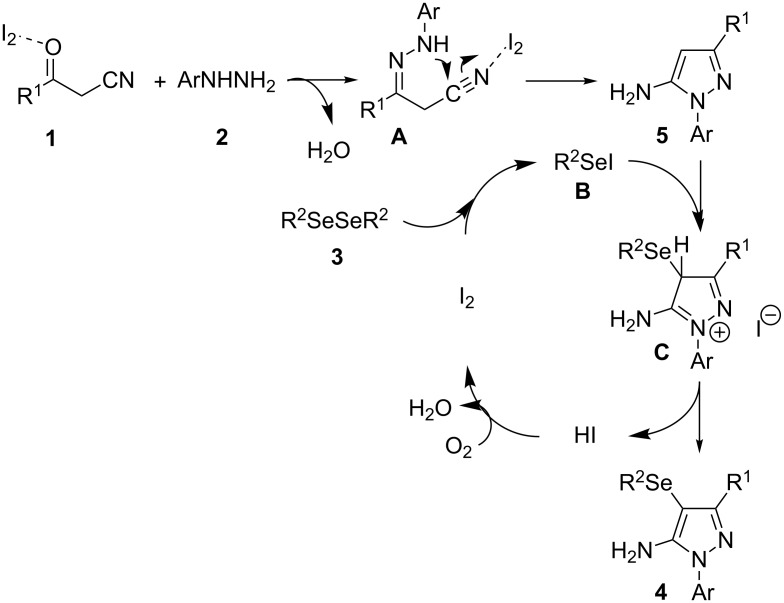
Proposed reaction mechanism.

On the basis of previous reports [21,25] a mechanism can be proposed for this reaction as depicted in Scheme 4. Initially, we believe that arylhydrazine **2** reacts with benzoylacetonitrile **1** by a 1,2-addition reaction in the presence of iodine as Lewis acid to generate the hydrazone intermediate **A**. Then, hydrazone **A** undergoes a cyclization reaction followed by an oxidative aromatization to yield 1*H*-pyrazol-5-amine **5**. At the same time, the diaryl diselenide **3** reacts with the molecular iodine to generate an electrophilic selenium species **B**. The following step is the nucleophilic attack of the electron-rich pyrazole **5** to species **B** to afford product **4** after releases a proton and produce HI. The presence of oxygen into the reaction system is expected to regenerate the iodine to continue the catalytic cycle.

**Scheme 5:**
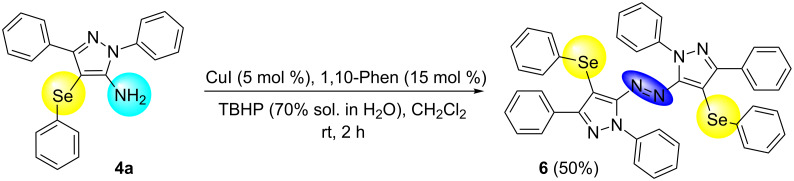
Synthesis of diazo pyrazole derivative **6**.

To verify the synthetic application of 5-amino-4-(arylselanyl)-1*H*-pirazoles **4**, we selected compound **4a** to prepare a new diazopyrazole derivative **6** based on the methodology described by Jiang and co-workers [26]. In this context, by reacting the 4-(phenylselanyl)pyrazol-5-amine **4a** with CuI (5 mol %), 1,10-phenantroline (15 mol %) and *tert-*butyl hydroperoxide in dichloromethane at room temperature for 2 h, the respective diazo aromatic compound **6** could be obtained in 50% yield (Scheme 5). As can be seen, the reaction proceeded smoothly allowing formation of the new N=N bond without cleavage of the C–Se bond under the oxidant media and the presence of copper salt.

**Figure 1:**
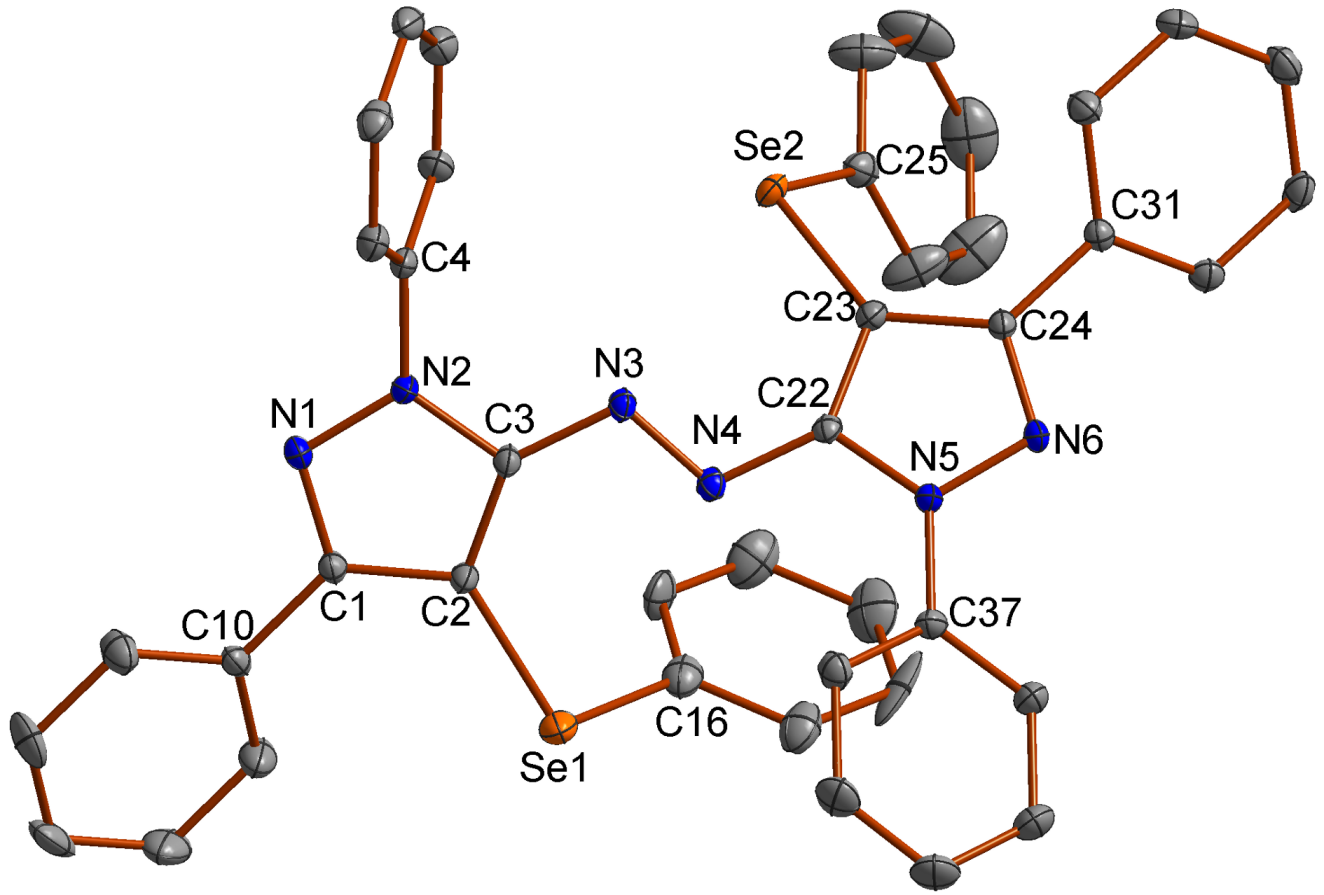
Molecular structure of compound **6**. The hydrogen atoms are omitted for clarity [27].

To confirm the structure of **6**, its molecular structure was also analyzed by single-crystal X-ray diffraction (CCDC: 1853770). The molecular structure is demonstrated in Figure 1.

## Conclusion

A variety of 4-(arylselanyl)-1*H*-pyrazol-5-amines was prepared in a one-pot multicomponent procedure starting from easily available benzoylacetonitriles, arylhydrazines and diaryl diselenides. Molecular iodine (50 mol %) was employed to promote this reaction in MeCN under reflux temperature and afforded the expected products in a range of 24% to 96% yield after 48 hours. Finally, the obtained 4-(phenylselanyl)-1*H*-pyrazol-5-amine proved to be a promising starting material for synthesizing new diazo compounds with higher added value. The protocol described here can be considered a valuable tool for the advance of the synthesis and pharmacological studies of selenium-containing pyrazoles and derivatives.

## Supporting Information

The Supporting Information features full experimental data and copies of ^1^H and ^13^C NMR spectra of pyrazoles **4** and **6**.

File 1 Experimental part.
