# Supplementary material for: Molecular iodine-catalyzed one-pot multicomponent synthesis of 5-amino-4-(arylselanyl)-1H-pyrazoles
Source: Beilstein J Org Chem. 2018 Nov 6;14:2789–98. doi: 10.3762/bjoc.14.256 (PMC6244180; doi:10.3762/bjoc.14.256)
Supplement: File 1 — Experimental part. [file Beilstein_J_Org_Chem-14-2789-s001.pdf]

## Supporting Informartion

## for

# Molecular iodine-catalyzed one-pot multicomponent synthesis of 5-amino-4-(arylselanyl)-1*H*-pyrazoles

Camila S. Pires[1], Daniela H. de Oliveira[1], Maria R. B. Pontel[1], Jean C. Kazmierczak[2], Roberta Cargnelutti[2], Diego Alves[1], Raquel G. Jacob[1] and Ricardo F. Schumacher*[1,2]

Address: [1]LASOL - CCQFA - Universidade Federal de Pelotas - UFPel - P.O. Box 354 - 96010-900, Pelotas, RS, Brazil and [2]Departamento de Química, Universidade Federal de Santa Maria – UFSM, 97105-900, Santa Maria, RS, Brazil

Email: Ricardo F. Schumacher - ricardo.schumacher@ufsm.br

*Corresponding author

## Experimental part

## Contents

**General Information ........................................................................ S2**

**General procedure for the synthesis of 5-amino-4-(arylselanyl)pyrazoles (4) ........................................................................................................ S2**

**General procedure for the synthesis of diazo pyrazole derivative (6) ….. S3**

**Table S1. Crystallographic data and refinement parameters for 6 ……… S8**

**Selected spectra …….......……………………………...……………............…… S9**

**General Information:** Reactions were monitored by TLC carried out on Merck silica gel (60 F254) by using UV light as visualizing agent and 5% vanillin in 10% $H_2SO_4$ and heat as developing agents. Column chromatography was performed by using silica gel. Proton nuclear magnetic resonance spectra ($^1$H NMR) were obtained at 400 MHz with Bruker DPX 400 spectrometer. Spectra were recorded in $CDCl_3$ solutions. Chemical shifts are reported in ppm, referenced to tetramethylsilane (TMS) as the external reference. Hydrogen coupling patterns are described as singlet (s), doublet (d), triplet (t), double doublet (dd) and multiplet (m). Coupling constants (*J*) are reported in Hertz. Carbon-13 nuclear magnetic resonance spectra ($^{13}$C NMR) were obtained at 100 MHz with Bruker DPX 400 spectrometer. Chemical shifts are reported in ppm, referenced to the solvent peak of $CDCl_3$ (77.0 ppm). Low resolution mass spectra (MS) were obtained with Shimadzu GCMS-QP2010 mass spectrometer 10416. High resolution mass spectra (HRMS) were recorded on a Bruker Micro TOF-QII spectrometer. Melting point (m.p.) values were measured with a Marte PFD III instrument. Infrared spectra were measured using a Shimadzu Prestige-21 IR spectrometer. A Cole Parmer-ultrasonic processor Model CPX 130, with a maximum power of 130 W, operating at amplitude of 60% and a frequency of 20 kHz was used.

### General procedure for the synthesis of 5-amino-4-(arylselanyl)pyrazoles (4)

To a 10 mL vial equipped with a reflux condenser containing a mixture of benzoylacetonitrile **1** (0.5 mmol), arylhydrazine **2** (0.7 mmol) and diaryl diselenide **3** (0.5 mmol) in MeCN (3 mL), molecular iodine (50 mol %) was added. Then the mixture was stirred at reflux temperature for a period of 48 hours. Under these conditions the expected product **4** was obtained and the progress of the reaction was monitored by TLC. The reaction mixture was poured in water (15 mL), extracted with ethyl acetate (3 × 10 mL), dried over $MgSO_4$ and concentrated under vacuum. The residue was purified by column

chromatography by using silica gel and a mixture of hexane/ethyl acetate (95:05) as eluent.

**General procedure for the synthesis of diazo pyrazole derivative (6)**[1]

5-Amino-4-(phenylselanyl)-1*H*-pyrazol **4a** (0.5 mmol) was added to a 10 mL vial containing a mixture of 1,10-phenantroline (0.15 mmol), *tert*-butyl peroxide (1.5 mmol) and CuI (0.05 mmol) in dichloromethane (2 mL). Then, the mixture was stirred at room temperature for 2 hours and the expected product **5** was obtained. The progress of the reaction was monitored by TLC. The reaction mixture was received in water (10 mL), extracted with ethyl acetate (3 × 5 mL), dried over $MgSO_4$ and concentrated under vacuum. The residue was purified by column chromatography using silica gel and a mixture of hexane/ethyl acetate (90:10) as eluent.

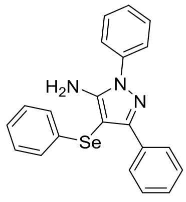


**1,3-Diphenyl-4-(phenylselanyl)-1*H*-pyrazol-5-amine (4a):** Orange oil; Yield: 96%; IR (KBr, $cm^{-1}$): 3450 (s), 3412 (s), 3055 (m), 2922 (w), 1946 (w), 1801 (w), 1600 (s), 1500 (s), 1475 (m), 1371 (m), 1111 (w), 970 (m), 759 (s), 690 (s). $^1$H NMR ($CDCl_3$, 400 MHz): $\delta$=7.93-7.91 (m, 2H), 7.67-7.65 (m, 2H), 7.50-7.46 (m, 2H), 7.37-7.25 (m, 6H), 7.21–7.13 (m, 3H) 4.30 ppm (s, 2H). $^{13}$C NMR ($CDCl_3$, 100 MHz): $\delta$=153.6, 149.5, 138.6, 133.0, 132.9, 129.5, 129.2, 128.1, 128.1, 127.9 127.8, 127.6, 125.8, 123.6, 82.4 ppm. $^{77}$Se NMR ($CDCl_3$, 76 MHz): $\delta$= 193.9 ppm. MS *m/z* (rel. int.): 391 (43), 389 (23), 311 (100), 293 (3), 208 (15), 155 (8). HRMS calcd. for $C_{21}H_{18}N_3Se$: $[M + H]^+$ 392.0660. Found: 392.0663.

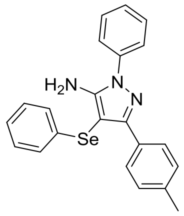


**1-Phenyl-4-(phenylselanyl)-3-(*p*-tolyl)-1*H*-pyrazol-5-amine (4b):** Reddish oil; Yield: 73%; IR (KBr, $cm^{-1}$): 3433 (s), 3057 (w), 2916 (w), 1598 (s), 1502 (s), 1473 (m), 1367 (w), 1182 (w), 1020 (m), 970 (m), 732 (s), 665 (w). $^1$H NMR ($CDCl_3$, 400 MHz): $\delta$=7.83-7.81 (m, 2H), 7.68–7.66 (m, 2H), 7.51-7.47 (m, 2H), 7.38-7.34 (m, 2H), 7.26-7.12 (m, 6H), 4.30 (s, 2H), 2.33 ppm (s, 3H). $^{13}$C NMR ($CDCl_3$, 100 MHz): $\delta$=153.7, 149.5, 137.9, 133.2, 130.6, 130.2, 129.6, 129.3, 128.9, 128.0 127.8, 127.6, 125.9, 123.7, 82.6, 21.2 ppm. $^{77}$Se NMR ($CDCl_3$, 76 MHz): $\delta$= 194.3 ppm. MS *m/z* (rel. int.): 405 (37), 325 (100), 307 (2), 232 (3), 208 (13), 162 (4), 132 (15), 119 (14), 77 (35). HRMS calcd. for $C_{22}H_{20}N_3Se$: $[M + H]^+$ 406.0817. Found: 406.0820.

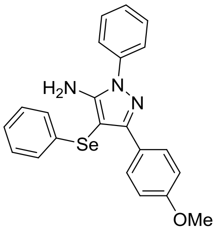


**3-(4-methoxyphenyl)-1-phenyl-4-(phenylselanyl)-1*H*-pyrazol-5-amine (4c):** Brownish oil; Yield: 35%; $^{1}$H NMR ($CDCl_3$, 400 MHz): *δ*= 7.89-7.85 (m, 2H), 7.66-7.63 (m, 2H), 7.48-7.44 (m, 2H), 7.34-7.30 (m, 1H), 7.26-7.24 (m, 2H), 7.21-7.16 (m, 2H), 7.14-7.10 (m, 1H), 6.87-6.83 (m, 2H), 4.28 (s, 2H), 3.75 ppm (s, 3H). $^{13}$C NMR ($CDCl_3$, 100 MHz): *δ*= 159.6, 153.3, 149.4, 138.7, 133.1, 129.5, 129.2, 129.0, 127.8, 127.4, 125.8, 125.6, 123.5, 113.5, 82.1, 55.1 ppm. MS *m/z* (rel. int.): 421 (1), 408 (1), 345 (4), 280 (61), 265 (51), 146 (47), 131 (23), 119 (47), 104 (49), 77 (100).

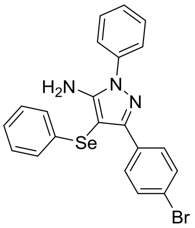


**3-(4-Bromophenyl)-1-phenyl-4-(phenylselanyl)-1*H*-pyrazol-5-amine (4d):** Brown solid; m.p.: 43-45 °C; Yield: 78%; IR (KBr, $cm^{-1}$): 3412 (m), 3055 (w), 2920 (w), 2850 (w), 1598 (s), 1575 (m), 1498 (s), 1436 (m), 1363 (m), 1008 (m), 912 (w), 829 (s), 665 (w). $^{1}$H NMR ($CDCl_3$, 400 MHz): *δ*=7.76-7.74 (m, 2H), 7.56-7.54 (m, 2H), 7.41-7.35 (m, 4H), 7.28-7.25 (m, 1H), 7.16-7.04 (m, 5H), 4.14 ppm (s, 2H). $^{13}$C NMR ($CDCl_3$, 100 MHz): *δ*=152.3, 149.7, 138.6, 132.8, 132.0, 131.2, 129.6, 129.3, 127.9, 127.8, 126.0, 123.6, 123.4, 82.5 ppm. $^{77}$Se NMR ($CDCl_3$, 76 MHz): *δ*= 192.9 ppm. MS *m/z* (rel. int.): [M+2] 471 (40) [M+] 469 (51), 389 (99), 309 (7), 267 (5), 233 (4), 208 (33), 155 (13), 127 (15), 119 (28), 77 (100). HRMS calcd. for $C_{21}H_{17}BrN_3Se$: $[M + H]^+$ 469.9766. Found: 469.9763.

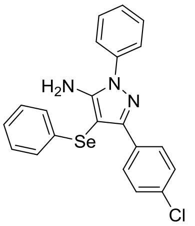


**3-(4-Chlorophenyl)-1-phenyl-4-(phenylselanyl)-1*H*-pyrazol-5-amine (4e):** Brown oil; Yield: 81%; IR (KBr, $cm^{-1}$): 3550 (m), 3414 (s), 3057 (m), 2920 (w), 1948 (w), 1884 (w), 1598 (s), 1531 (m), 1500 (s), 1454 (m), 1373 (m), 1087 (s), 759 (s), 659 (w). $^{1}$H NMR ($CDCl_3$, 400 MHz): *δ*=7.83-7.81 (m, 2H), 7.58–7.56 (m, 2H), 7.41 (t, *J*=7.4 Hz, 2H), 7.30-7.26 (m, 1H), 7.22-7.20 (m, 2H), 7.17-7.06 (m, 5H), 4.18 ppm (s, 2H). $^{13}$C NMR ($CDCl_3$, 100 MHz): *δ*=152.4, 149.6, 138.6, 134.1, 132.8, 131.6, 129.6, 129.3, 129.1, 128.3, 128.0, 127.8, 126.1, 123.7, 82.5 ppm. MS *m/z* (rel. int.): [M+2] 427 (10) 425 (31), 421 (6), 347 (35), 345 (100), 309 (4), 267 (3), 233 (2), 208 (16), 180 (2), 162 (9), 153 (3), 177 (35). HRMS calcd. for $C_{21}H_{17}ClN_3Se$: $[M + H]^+$ 426.0271. Found: 426.0262.

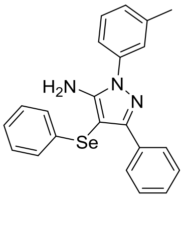


**3-Phenyl-4-(phenylselanyl)-1-(*m*-tolyl)-1*H*-pyrazol-5-amine (4g):** Brown oil; Yield: 63%; IR (KBr, $cm^{-1}$): 3473 (m), 3408 (s),

3331 (m), 3097 (w), 2922 (w), 2852 (w), 1606 (s), 1575 (m), 1541 (s), 1496 (m), 1346 (s), 1109 (w), 966 (m), 742 (s), 657 (w). $^1$H NMR ($CDCl_3$, 400 MHz): $\delta$=7.92-7.90 (m, 2H), 7.48-7.12 (m, 12H), 4.28 (s, 2H), 2.39 ppm (s, 3H). $^{13}$C NMR ($CDCl_3$, 100 MHz): $\delta$=153.4, 149.4, 139.8, 138.4, 133.1, 132.9, 129.2, 128.4, 128.1, 127.8, 127.7, 125.8, 124.8, 120.4, 82.1, 21.3 ppm. MS *m/z* (rel. int.): 405 (31), 391 (3), 325 (100), 283 (17), 248 (3), 222 (15), 146 (17). HRMS calcd. for $C_{22}H_{20}N_3Se$: $[M + H]^+$ 406.0817. Found: 406.0818.

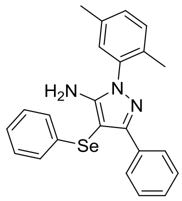


**1-(2,5-Dimethylphenyl)-3-phenyl-4-(phenylselanyl)-1*H*-pyrazol-5-amine (4h):** Orange oil; Yield: 52%; IR (KBr, cm$^{-1}$): 3550 (s), 3415 (s), 3055 (w), 2920 (w), 2854 (w), 1616 (s), 1508 (w), 1367 (w), 1143 (w), 968 (m), 815 (m), 771 (m), 734 (m), 694 (m). $^1$H NMR ($CDCl_3$, 400 MHz): $\delta$=7.84 (d, *J*=8.1 Hz, 2H), 7.24-7.16 (m, 6H), 7.12-7.01 (m, 5H), 3.90 (s, 2H), 2.28 (s, 3H), 2.09 ppm (s, 3H). $^{13}$C NMR ($CDCl_3$, 100 MHz): $\delta$=153.1, 150.3, 139.7, 136.2, 134.2, 133.5, 133.3, 132.0, 129.2, 128.0, 127.9, 127.8, 127.7, 127.6, 127.5, 125.7, 80.3, 21.1, 17.4 ppm. MS *m/z* (rel. int.): 419 (85), 417 (45), 339 (100), 323 (16), 297 (5), 2,62 (12), 235 (38), 184 (6), 159 (15). HRMS calcd. for $C_{23}H_{22}N_3Se$: $[M + H]^+$ 420.0973. Found: 420.0982.

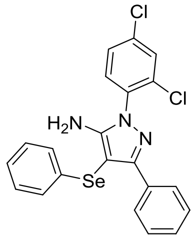


**1-(2,4-Dichlorophenyl)-3-phenyl-4-(phenylselanyl)-1*H*-pyrazol-5-amine (4i):** Brown oil; Yield: 80%; IR (KBr, cm$^{-1}$): 3550 (m), 3390 (s), 3248 (m), 3082 (s), 2915 (w), 2848 (w), 2362 (w), 1618 (s), 1537 (s), 1504 (s), 1469 (s), 1431 (m), 1296 (m), 1099 (m), 775 (s), 657 (m). $^1$H NMR ($CDCl_3$, 400 MHz): $\delta$=7.82-7.80 (m, 2H), 7.49-7.42 (m, 2H), 7.32-7.05 (m, 9H), 4.02 ppm (s, 2H). $^{13}$C NMR ($CDCl_3$, 100 MHz): $\delta$=154.5, 150.9, 136.2, 134.6, 133.0, 132.9, 132.8, 130.8, 130.4, 129.3, 128.4, 128.3, 128.1, 127.9, 127.8, 125.9, 82.0 ppm. MS *m/z* (rel. int.): [M+2] 461 (37), [M+] 459 (55), 424 (100), 381 (50), 321 (8), 276 (17), 218 (11), 199 (14), 128 (41), 77 (60). HRMS calcd. for $C_{21}H_{16}Cl_2N_3Se$: $[M + H]^+$ 459.9881. Found: 459.9882.

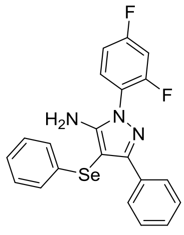


**1-(2,4-Difluorophenyl)-3-phenyl-4-(phenylselanyl)-1*H*-pyrazol-5-amine (4j):** Brown solid; m.p.: 102-103 °C; Yield: 76%; IR (KBr, cm$^{-1}$): 3550 (m), 3473 (s), 3412 (s), 3230 (w), 3051 (w), 2920 (w), 1637 (m), 1616 (s), 1597 (s), 1531 (m), 1363 (w), 831 (s), 723 (m), 620 (w). $^1$H NMR ($CDCl_3$, 400 MHz): $\delta$=7.90-7.87 (m, 2H), 7.68-7.62 (m, 1H), 7.37-7.30 (m, 3H), 7.27-7.20 (m, 4H), 7.18-7.15 (m, 1H), 7.08-7.01 (m, 2H), 4.22 ppm (s, 2H). $^{13}$C NMR ($CDCl_3$, 100 MHz): $\delta$=162.5 (dd, *J*=241.2 e 11.1 Hz), 156.7 (dd, *J*=241.2 e 11.1 Hz), 154.7, 151.0, 147.9, 143.4, 132.9, 132.7, 130.0, 129.9, 129.3, 128.3, 128.1, 127.8,

127.7, 127.1, 125.9, 122.6 (dd, *J*= 12.1, 4.0 Hz), 112.5 (dd, *J*= 22.5, 3.7 Hz), 105.1 (dd, *J*=26.5, 23.6 Hz), 82.3 ppm. $^{77}$Se NMR ($CDCl_3$, 76 MHz): $\delta$= 195.2 ppm. MS *m/z* (rel. int.): 427 (48), 425 (25), 383 (2), 347 (100), 305 (2), 244 (23), 218 (6), 155 (19), 128 (18). HRMS calcd. for $C_{21}H_{16}F_2N_3Se$: $[M + H]^+$ 428.0472. Found: 428.0486.

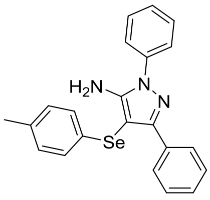


**1,3-Diphenyl-4-(*p*-tolylselanyl)-1*H*-pyrazol-5-amine (4l):** Orange oil; Yield: 54%; IR (KBr, $cm^{-1}$): 3550 (m), 3477 (m), 3390 (s), 3250 (s), 3109 (s), 1881 (w), 1811 (w), 1743 (w), 1618 (s), 1575 (m), 1469 (s), 1431 (s), 1294 (m), 1139 (m), 1099 (s), 974 (s), 657 (m). $^{1}$H NMR ($CDCl_3$, 400 MHz): $\delta$=7.94-7.92 (m, 2H), 7.65-7.63 (m, 2H), 7.47 (t, *J*=7.5 Hz, 2H), 7.35-7.28 (m, 4H), 7.15 (d, *J*=8.0 Hz, 2H), 7.01 (d, *J*=8.0 Hz, 2H), 4.28 (s, 2H), 2.25 ppm (s, 3H). $^{13}$C NMR ($CDCl_3$, 100 MHz): $\delta$=153.5, 149.4, 138.6, 135.6, 132.9, 130.0, 129.5, 129.1, 128.1, 128.0, 127.9, 127.8, 127.5, 123.5, 82.7, 20.9 ppm. MS *m/z* (rel. int.): 405 (10), 328 (100), 312 (12), 298 (22), 142 (16), 77 (61). HRMS cald. for $C_{22}H_{20}N_3Se$: $[M + H]^+$ 406.0817. Found 406.0822.

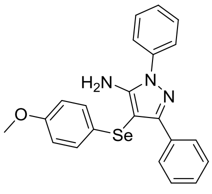


**4-((4-Methoxyphenyl)selanyl)-1,3-diphenyl-1*H*-pyrazol-5-amine (4m):** Brown solid; m.p.: 38-40 °C; Yield: 63%; IR (KBr, $cm^{-1}$): 3552 (w), 3442 (m), 3342 (m), 3053 (w), 2920 (w), 1884 (w), 1734 (w), 1602 (s), 1529 (m), 1600 (s), 1471 (s), 1452 (m), 1384 (m), 1170 (w), 1008 (s), 688 (s). $^{1}$H NMR ($CDCl_3$, 400 MHz): $\delta$= 7.86-7.84 (m, 2H), 7.49-7.48 (m, 2H), 7.29 (t, *J*=7.3 Hz, 2H), 7.22-7.16 (m, 4H), 7.06 (d, *J*=7.0 Hz, 2H), 6.60 (d, *J*=6.9 Hz, 2 H), 4.16 (s, 2H), 3.53 ppm (s, 3H). $^{13}$C NMR ($CDCl_3$, 100 MHz): $\delta$= 158.4, 153.1, 149.3, 138.7, 133.1, 132.8, 130.0, 129.3, 127.9, 127.8, 127.3, 123.4, 122.8, 115.0, 83.7, 55.1 ppm. MS *m/z* (rel. int.): 421 (6), 347 (34), 345 (100), 327 (7), 309 (8), 281 (28), 267 (8), 253 (16), 235 (7), 207 (84), 191 (12), 155 (5), 119 (25), 104, (16), 77 (82). HRMS calcd. for $C_{22}H_{20}N_3OSe$: $[M + H]^+$ 422.0766. Found: 422.0791.

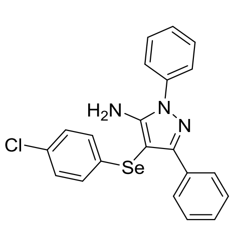


**4-((4-Chlorophenyl)selanyl)-1,3-diphenyl-1*H*-pyrazol-5-amine (4n):** Reddish solid; m.p.: 41-43 °C; Yield: 80%; IR (KBr, $cm^{-1}$): 3435 (s), 3059 (m), 2924 (w), 2850 (w), 2733 (w), 2194 (w), 1948 (w), 1884 (w), 1600 (s), 1531 (m), 1500 (s), 1454 (m), 1373 (m), 1174 (w), 1087 (s), 759 (s), 659 (w). $^{1}$H NMR ($CDCl_3$, 400 MHz): $\delta$= 7.82 (d, *J*=7.9 Hz, 2H), 7.56 (d, *J*=7.5 Hz, 2H), 7.39 (t, *J*=7.7 Hz, 2H), 7.26-7.22 (m, 4H), 7.10-7.04 (m, 4H), 6.84-6.64 (m, 1H), 4.22 ppm (s, 2H). $^{13}$C NMR ($CDCl_3$, 100 MHz) $\delta$= 153.5, 149.5, 138.6, 132.9, 131.9, 131.4, 129.6, 129.3, 129.2, 128.2, 128.1, 127.8, 123.6, 113.8, 82.2 ppm. $^{77}$Se NMR ($CDCl_3$, 76 MHz): $\delta$= 196.8

ppm. MS *m/z* (rel. int.): [M+2] 427 (19), [M+] 425 (42), 386 (13), 345 (100), 306 (44), 267 (3), 242 (11), 217 (8), 119 (20). HRMS cald. for $C_{21}H_{17}ClN_3Se$: $[M + H]^+$ 426.0271. Found: 426.0276.

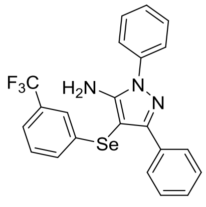


**4-((3-(trifluoromethyl)phenyl)selanyl)-1,3-diphenyl-1H-pyrazol-5-amine (4o):** Reddish oil; Yield: 24%; $^1$H NMR ($CDCl_3$, 400 MHz): $\delta$ = 7.09-6.96 (m, 2H), 6.69-6.33 (m, 12H), 3.38 ppm (s, 2H). $^{13}$C NMR ($CDCl_3$, 100 MHz): $\delta$ = 153.3, 149.5, 138.3, 134.5, 132.7, 131.8, 131.3 (d, *J* = 32 Hz), 130.9 (d, *J* = 1.5 Hz), 129.5, 128.2, 128.1, 127.7, 124.4 (d, *J* = 3.9 Hz), 123.6 (d, *J* = 272 Hz) 123.5, 122.6 (d, *J* = 3.9 Hz), 81.5 ppm. MS *m/z* (rel. int.): 459 (44), 457 (22), 379 (100), 358 (3), 341 (4), 281 (12), 253 (5), 207 (35), 180 (3), 132 (24), 119 (22), 77 (57).

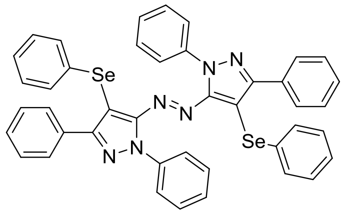


**(*E*)-1,2-bis(1,3-diphenyl-4-(phenylselanyl)-1*H*-pyrazol-5-yl)diazene (6):** Reddish solid; m.p.: 190-193 °C; Yield: 50%; $^1$H NMR ($CDCl_3$, 400 MHz): $\delta$= 7.85-7.83 (m, 4H), 7.57-7.36 (m, 24H), 7.30-7.26 ppm (m, 2H). $^{13}$C NMR ($CDCl_3$, 100 MHz): $\delta$= 151.1, 138.8, 131.6, 130.9, 129.6, 129.4, 129.3, 129.2, 129.2, 128.9, 128.7, 128.4, 128.2, 125.9, 125.8, 124.9, 124.5, 103.9. MS *m/z* (rel. int.): 778 (3), 621 (10), 541 (30), 311 (13), 169 (10), 155 (11), 91 (16), 77 (100).

**Reference:**


1. Jiang, B.; Ning, Y.; Fan, W.; Tu, S. J.; Li, G. *J. Org. Chem.* **2014**, *79*, 4018–4024.

**Table S1.** Crystallographic data and refinement parameters for **6**.

| Crystal Data | **6** |
|---|---|
| Formula | $C_{42}H_{30}N_6Se_2$ |
| Fw (g mol$^{-1}$) | 776.64 |
| *T* (K) | 100(2) |
| Crystal system | Monoclinic |
| Space group | $P2_1/c$ |
| *a*/Å | 12.6802(4) |
| *b*/Å | 32.3059(11) |
| *c*/Å | 8.5778(3) |
| α/° | 90 |
| *β*/° | 105.8660(10) |
| γ/° | 90 |
| *V*/Å$^3$ | 3380.0(2) |
| *Z* | 4 |
| $D_{calc}$ (g cm$^{-3}$) | 1.526 |
| *μ* (Mo Kα) (mm$^{-1}$) | 2.230 |
| *λ*/Å | 0.71073 |
| *F* (000) | 1568 |
| Collected reflns. | 57449 |
| Unique reflns. | 10347 |
| *Goof* ($F^2$) | 1.065 |
| $R_1$[a] | 0.0548 |
| $wR_2$[b] | 0.1227 |

[a] $R_1 = \sum ||F_o| - |F_c|| / \sum |F_o|$.

[b] $wR_2 = \{\sum w(F_o^2 - F_c^2)^2 / \sum w(F_o^2)^2\}^{1/2}$.

**SELECTED SPECTRA**

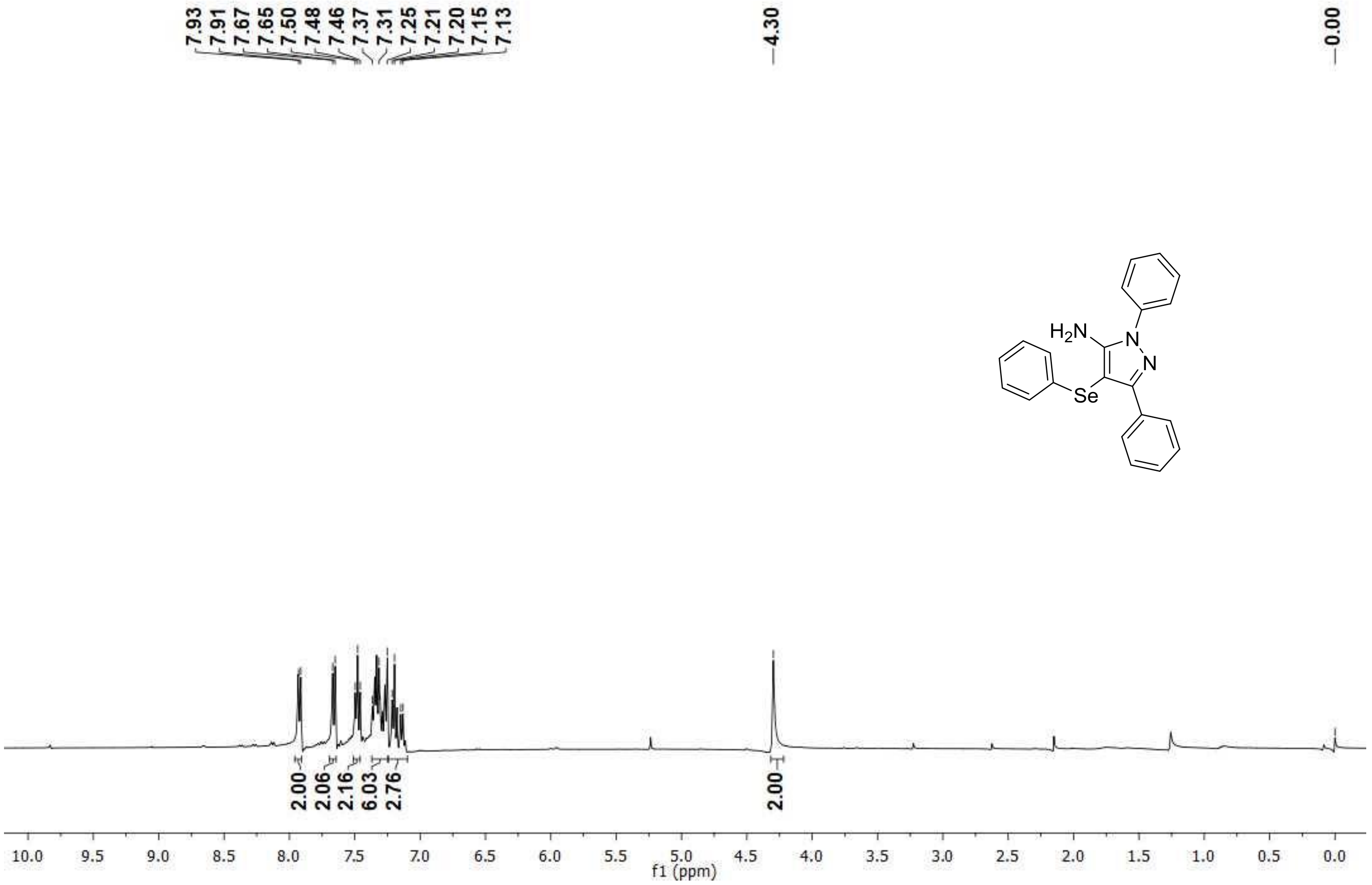


**Figure S1.** $^{1}$H NMR (400 MHz, $CDCl_3$) of the product **4a**.

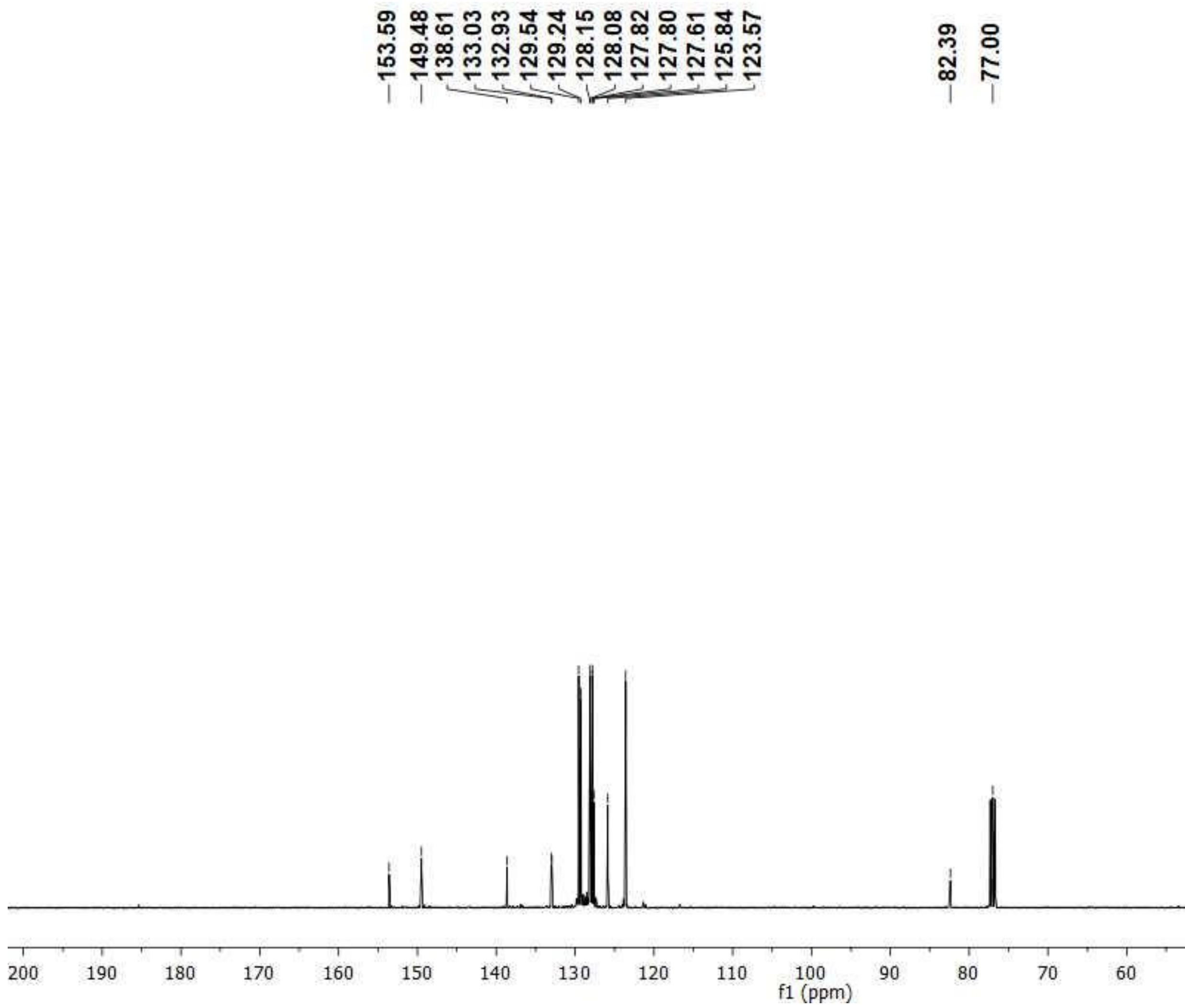


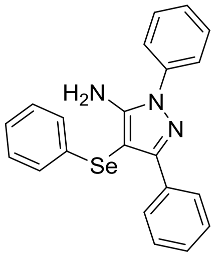


**Figure S2.** $^{13}$C NMR (100 MHz, $CDCl_3$) of the product **4a**.

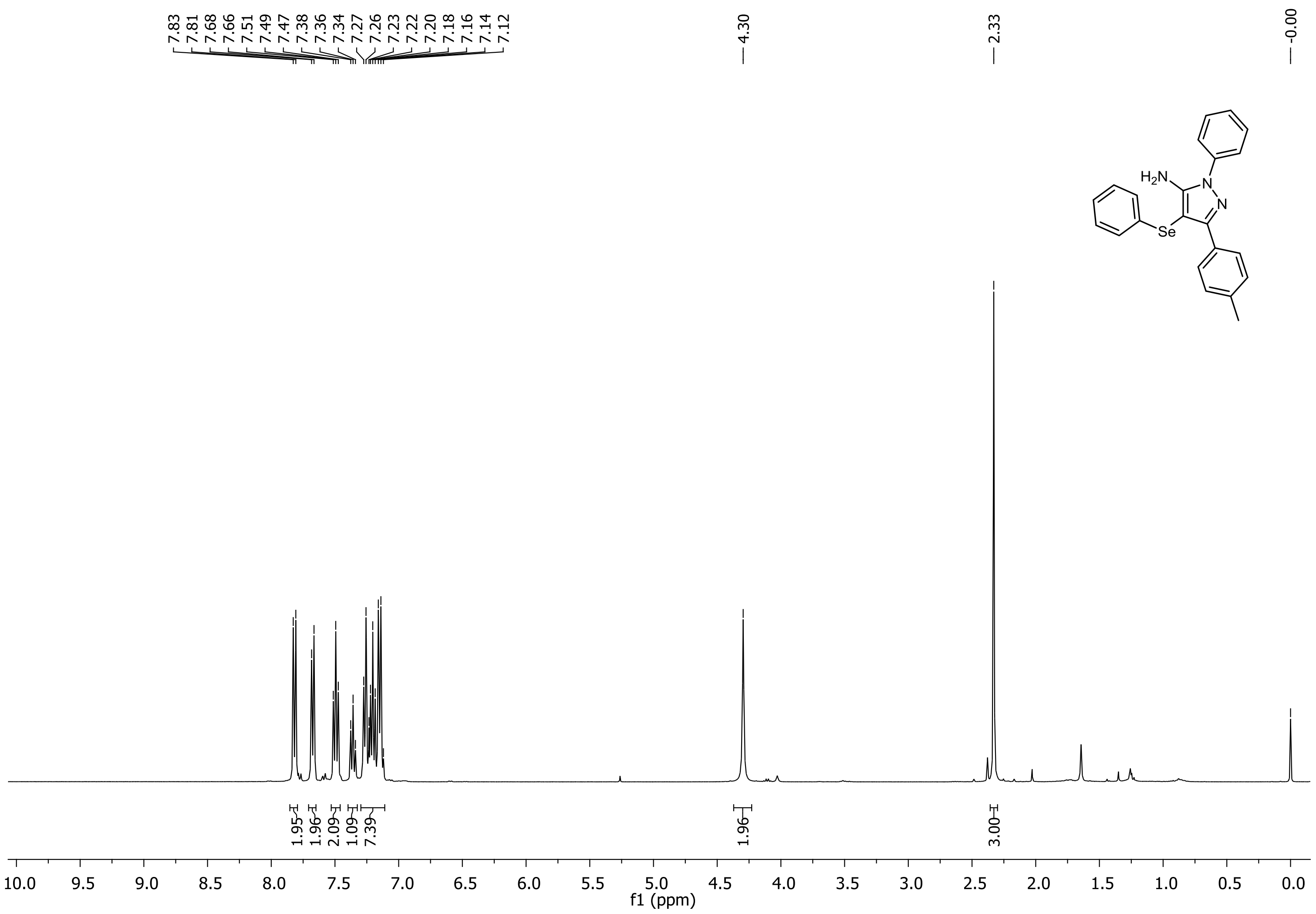


**Figure S3.** $^1$H NMR (400 MHz, $CDCl_3$) of the product **4b**.

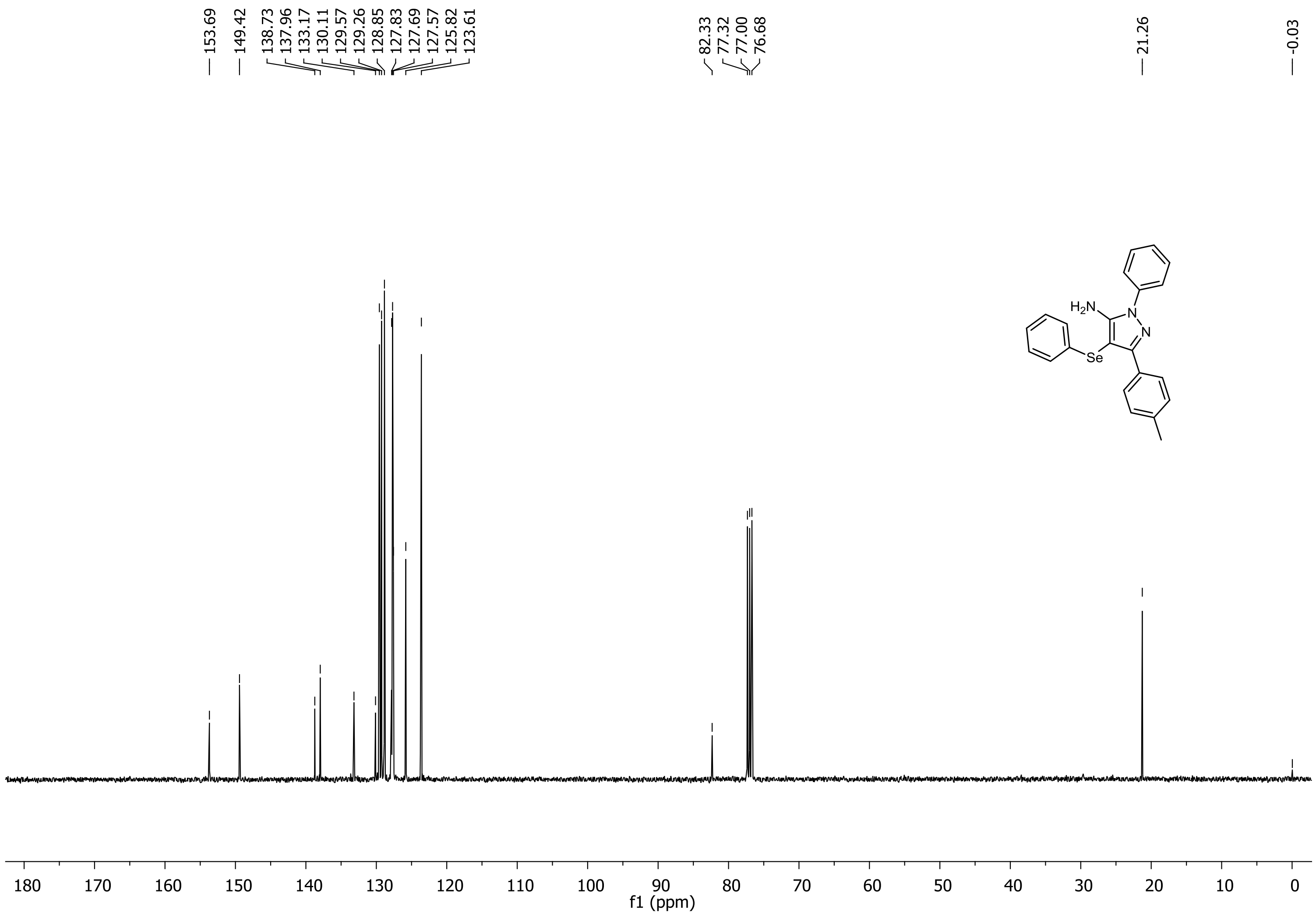


**Figure S4.** $^{13}C$ NMR (100 MHz, $CDCl_3$) of the product **4b**.

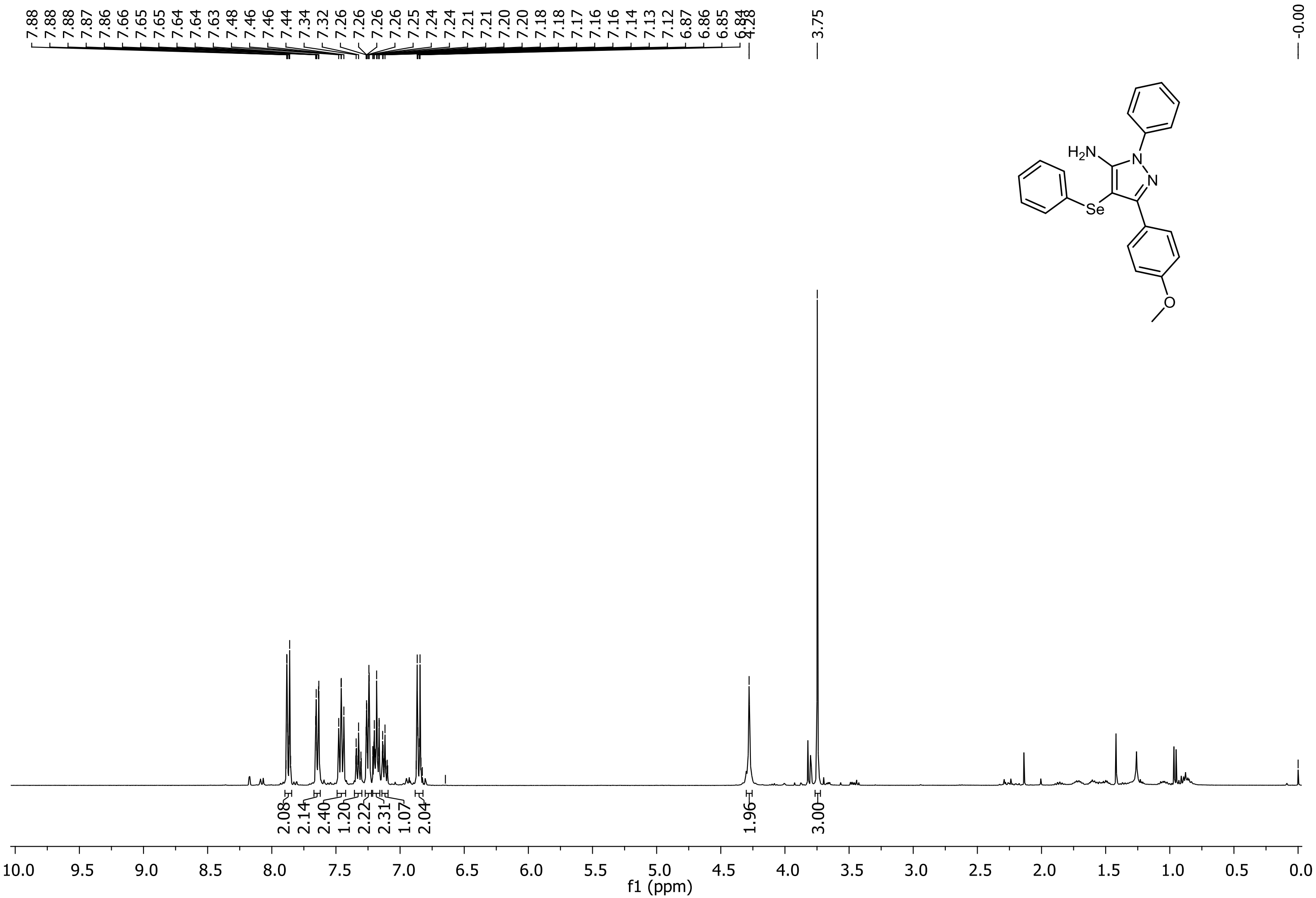

**Figure S5.** $^{1}$H NMR (400 MHz, $CDCl_3$) of the product **4b**.

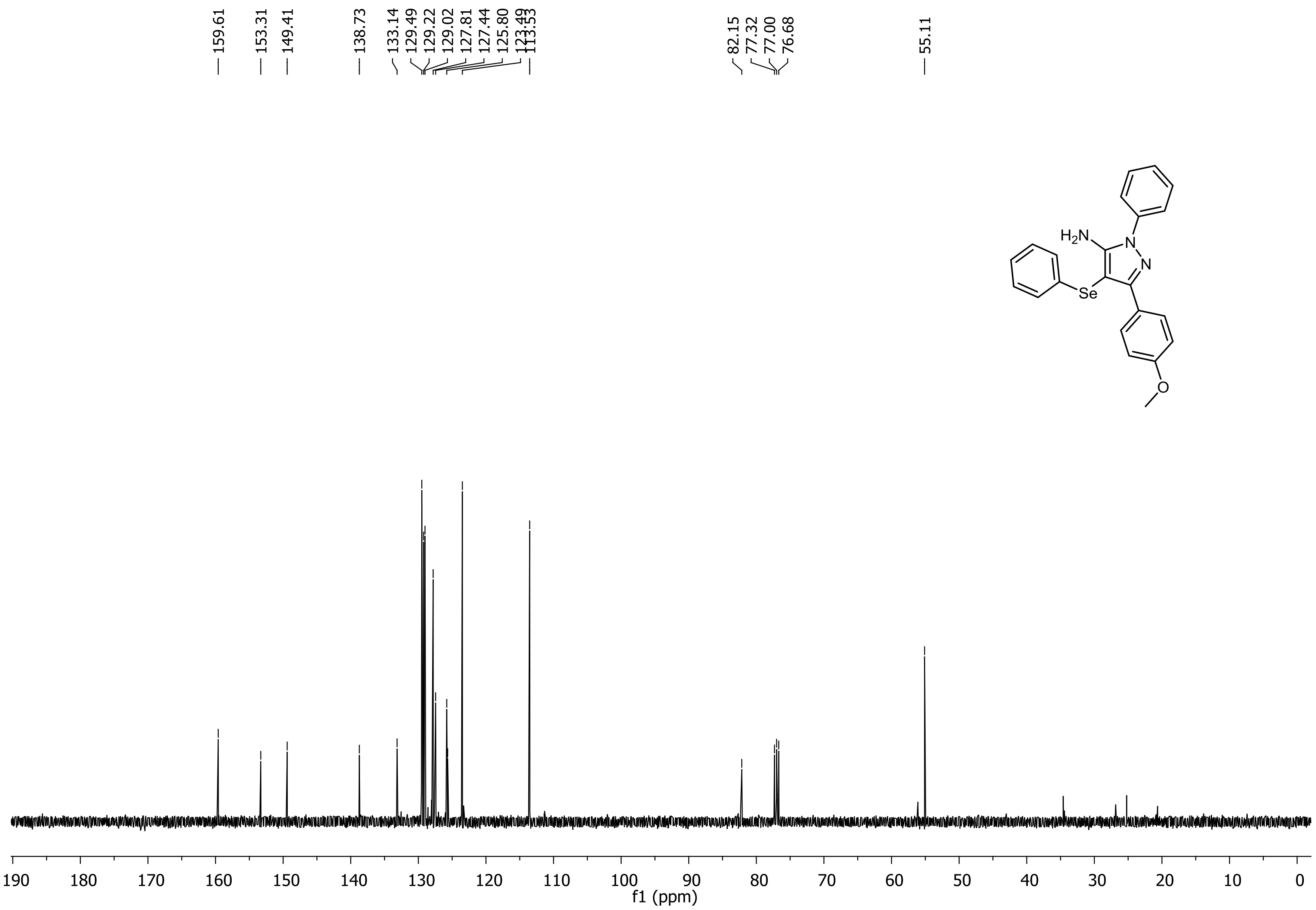


**Figure S6.** $^{13}$C NMR (100 MHz, $CDCl_3$) of the product **4c**.

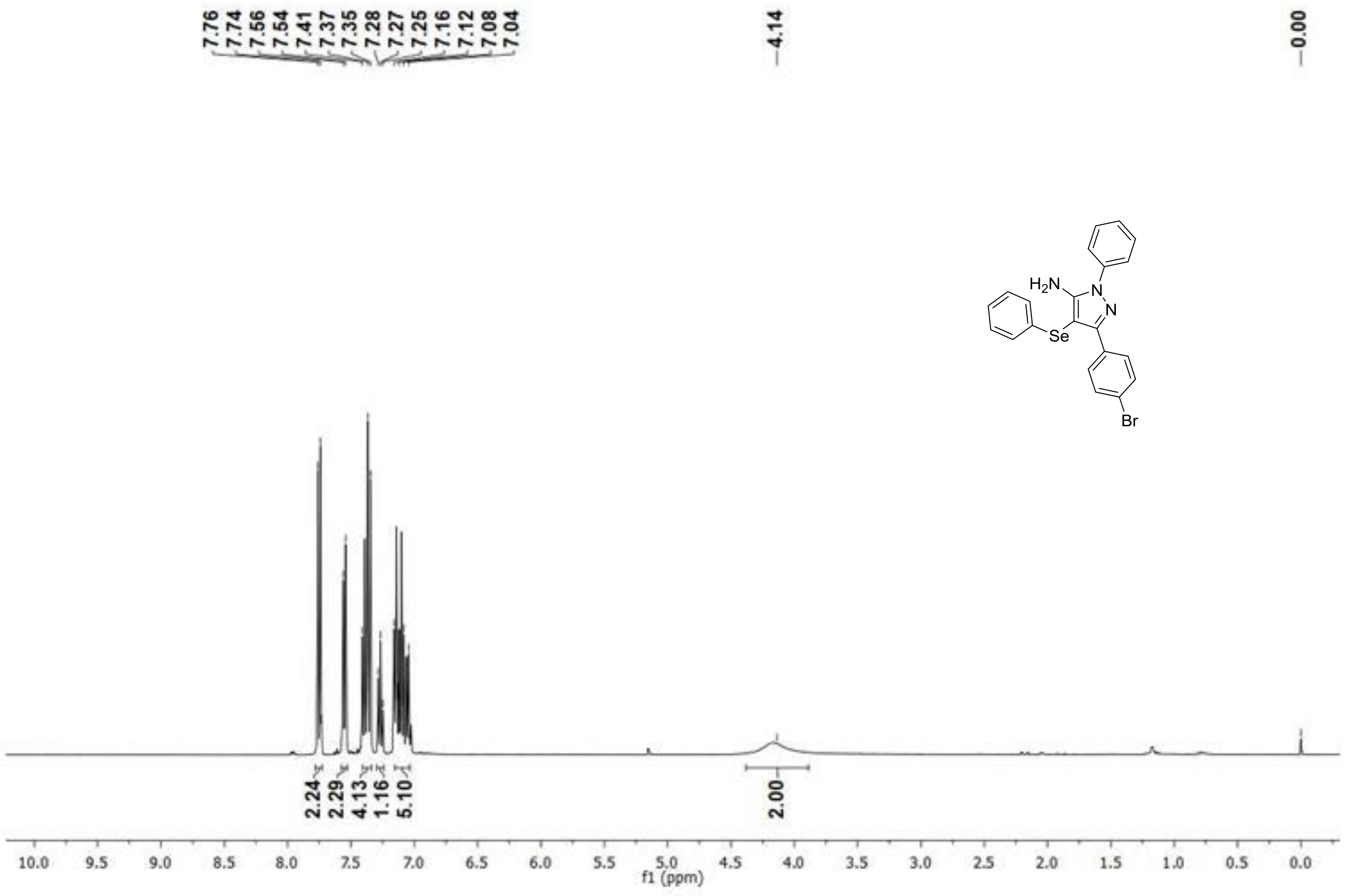


**Figure S7.** $^1$H NMR (400 MHz, $CDCl_3$) of the product **4d**.

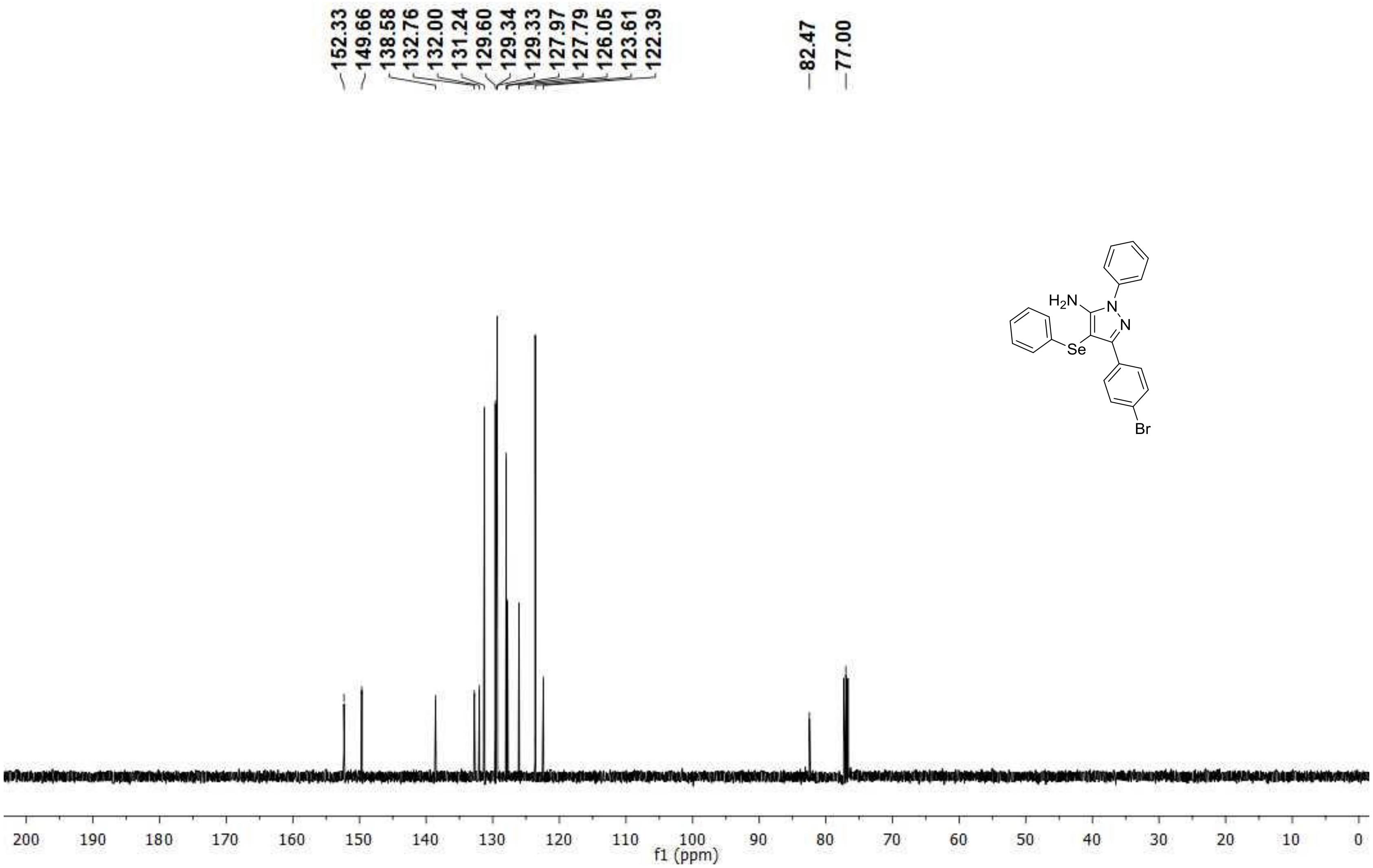


**Figure S8.** $^{13}C$ NMR (100 MHz, $CDCl_3$) of the product **4d**.

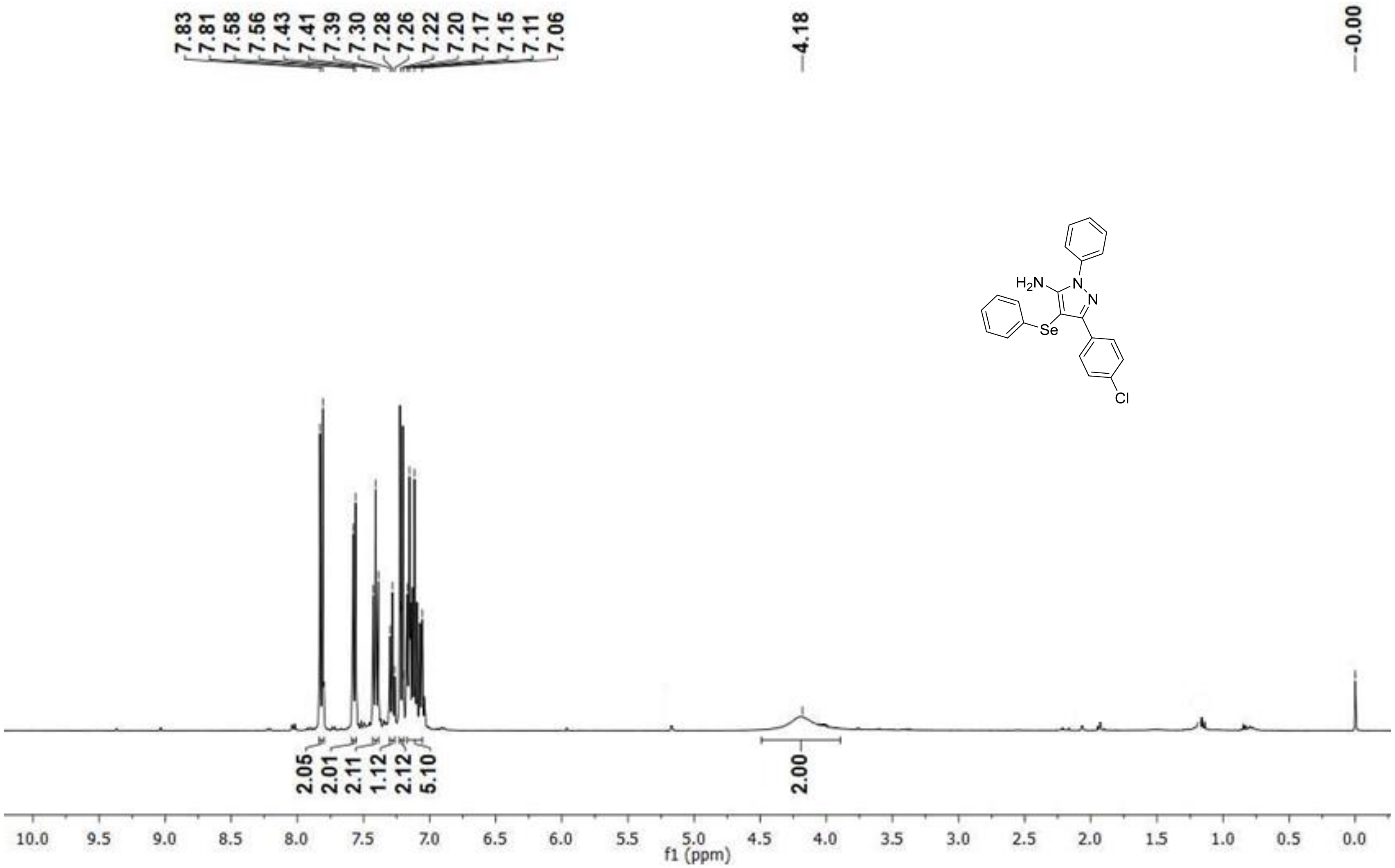


**Figure S9.** $^{1}$H NMR (400 MHz, $CDCl_3$) of the product **4e**.

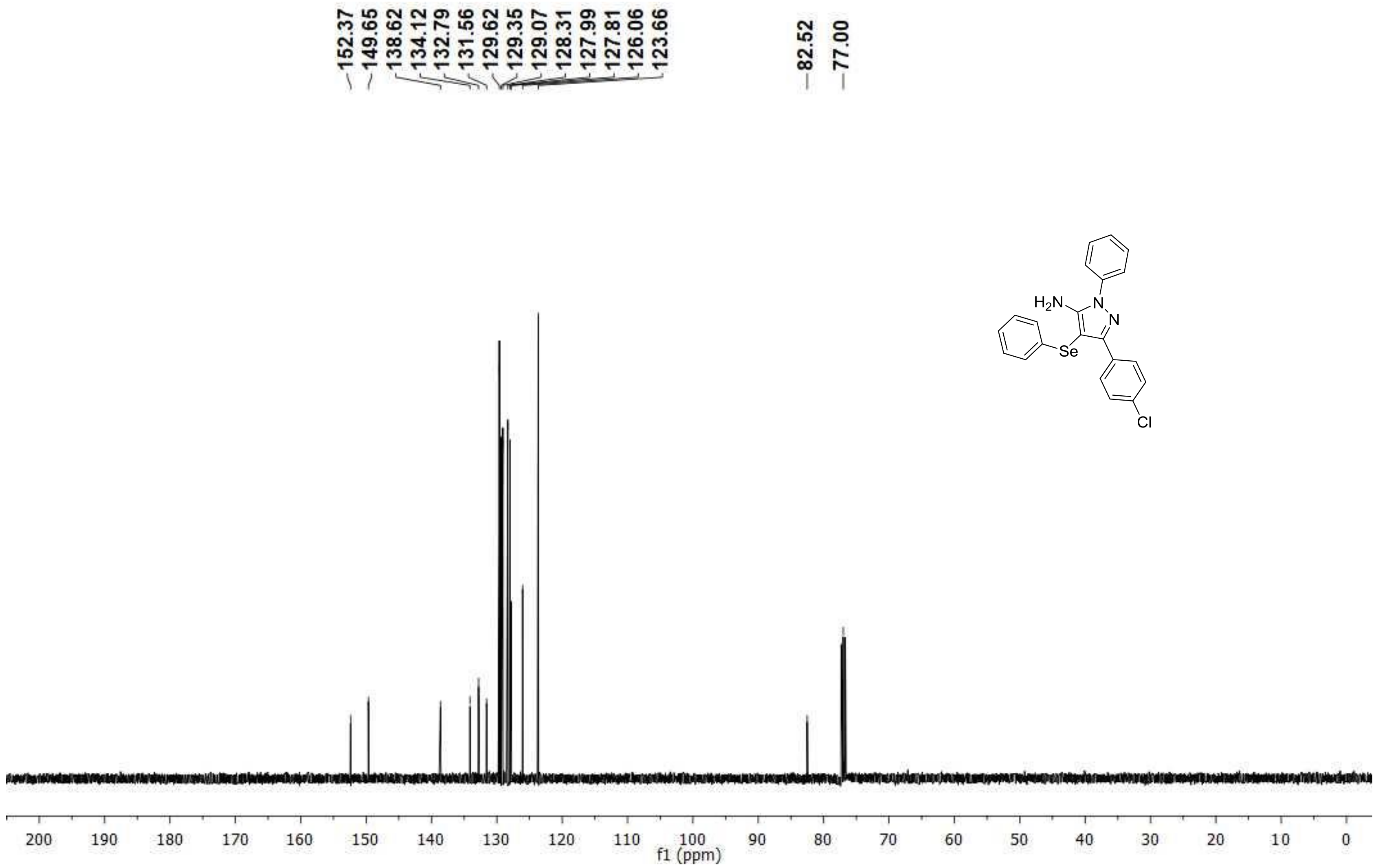


**Figure S10.** $^{13}$C NMR (100 MHz, $CDCl_3$) of the product **4e**.

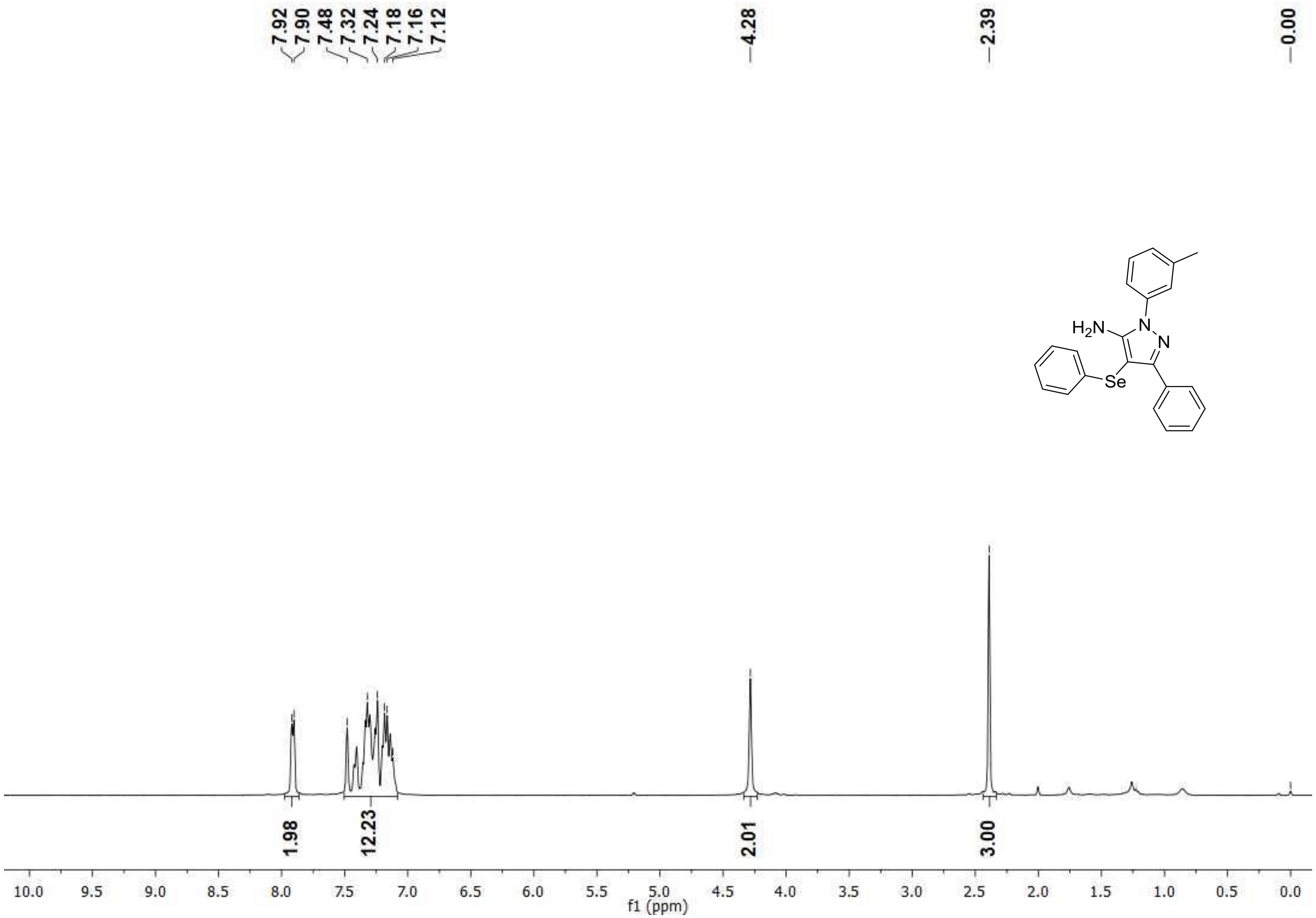


**Figure S11.** $^{1}$H NMR (400 MHz, $CDCl_3$) of the product **4g**.

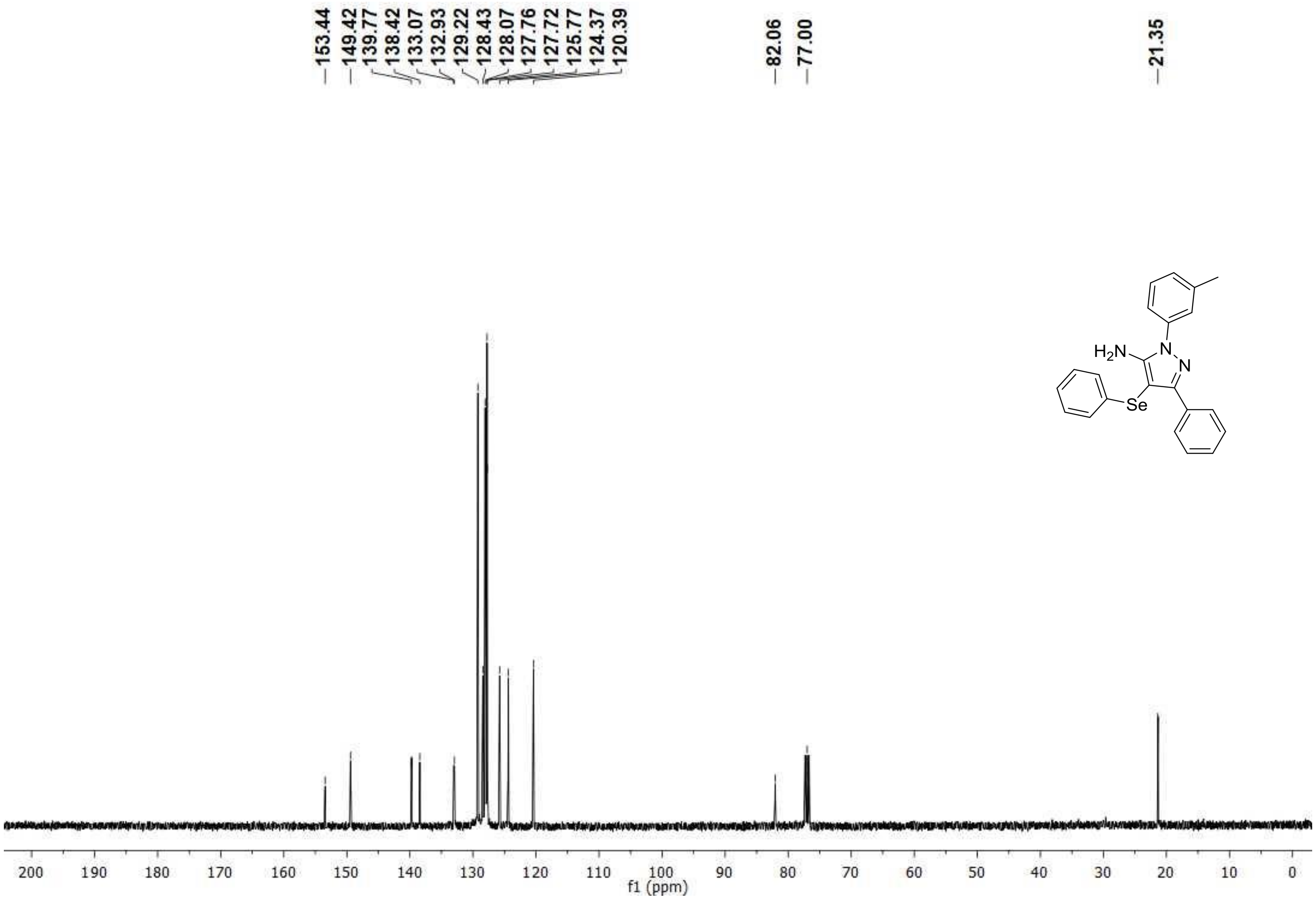


**Figure S12.** $^{13}C$ NMR (100 MHz, $CDCl_3$) of the product **4g**.

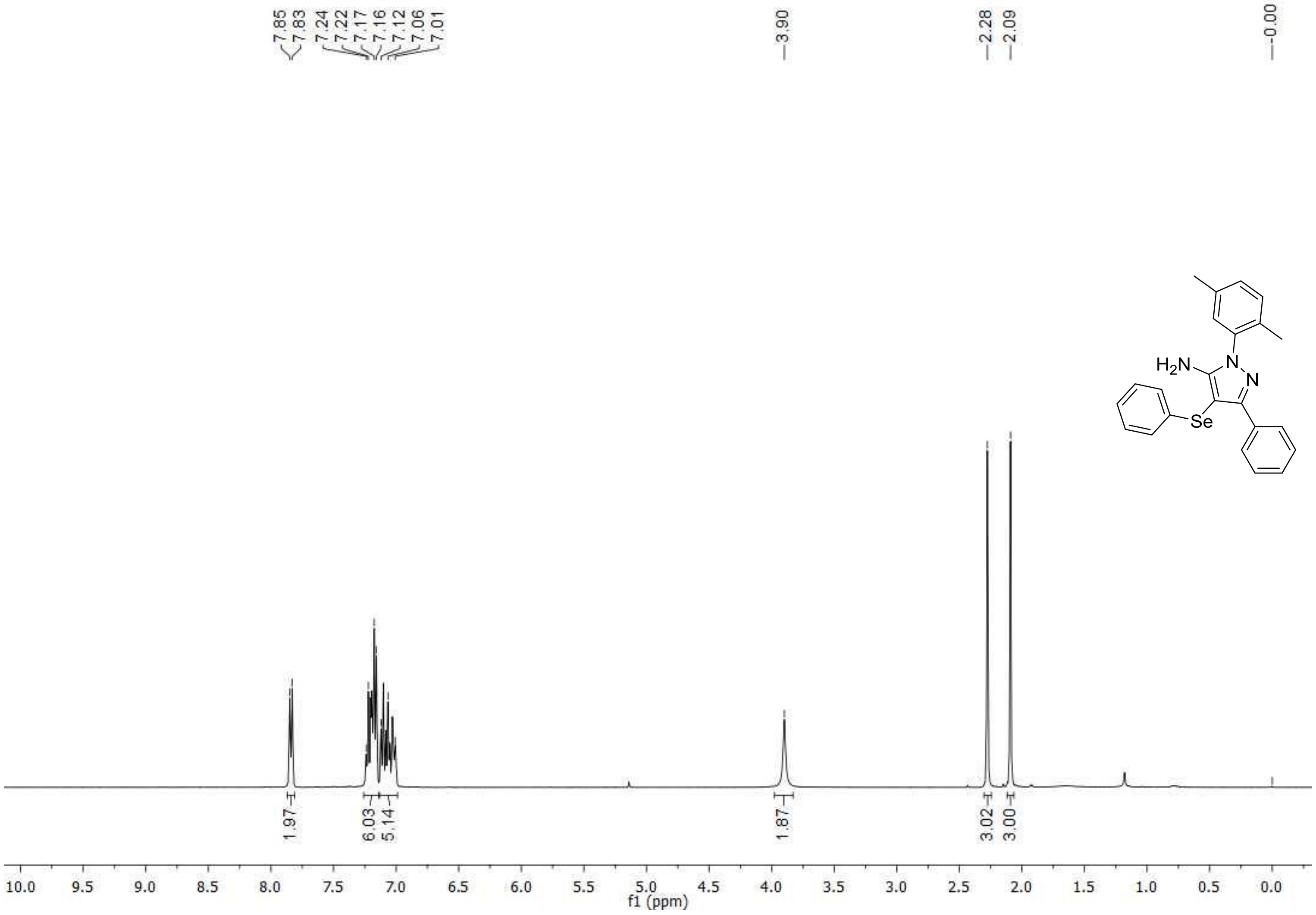


**Figure S13.** $^{1}$H NMR (400 MHz, $CDCl_3$) of the product **4h**.

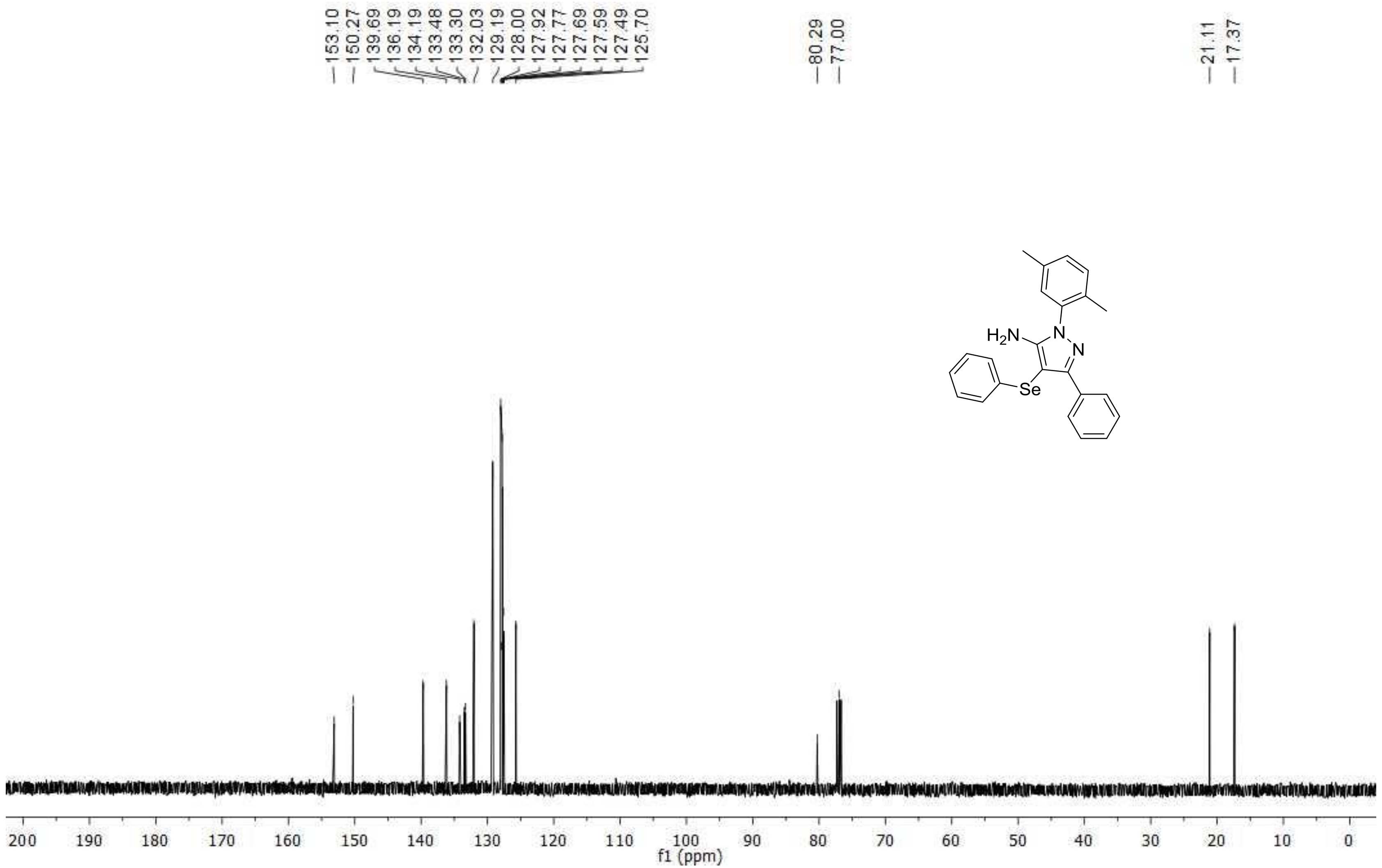


**Figure S14.** $^{13}$C NMR (100 MHz, $CDCl_3$) of the product **4h**.

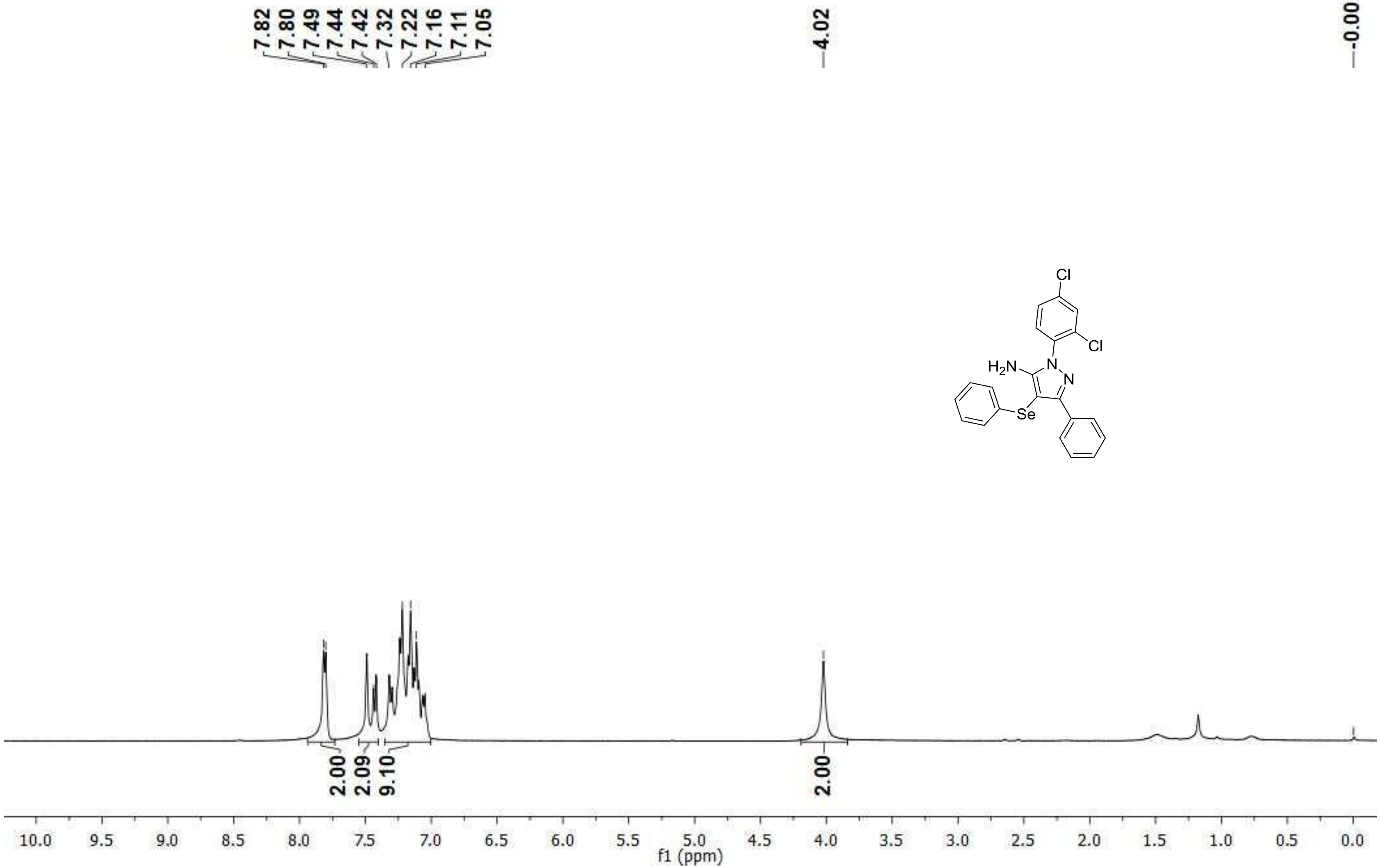


**Figure S15.** $^{1}$H NMR (400 MHz, $CDCl_3$) of the product **4i**.

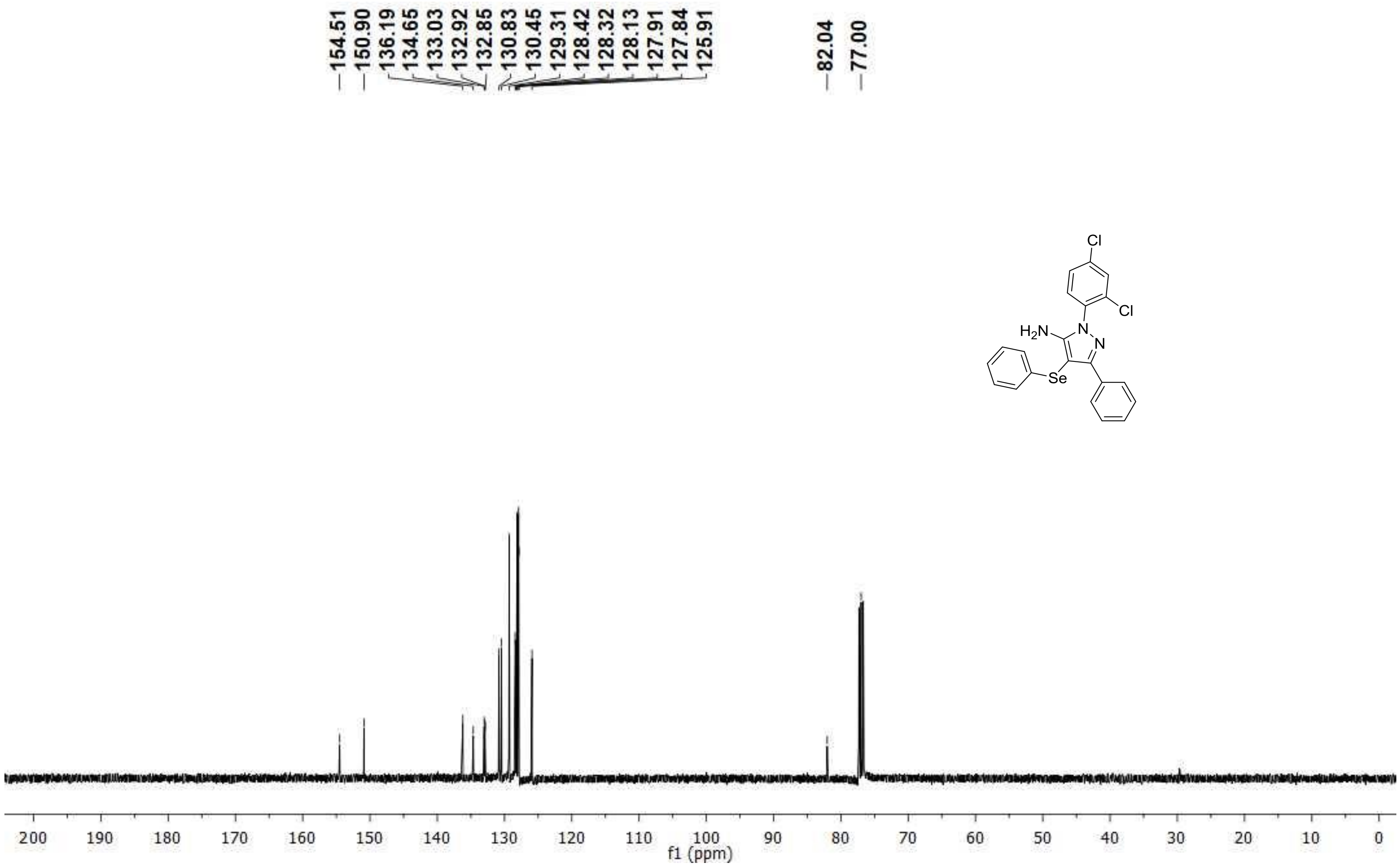


**Figure S16.** $^{13}C$ NMR (100 MHz, $CDCl_3$) of the product **4i**.

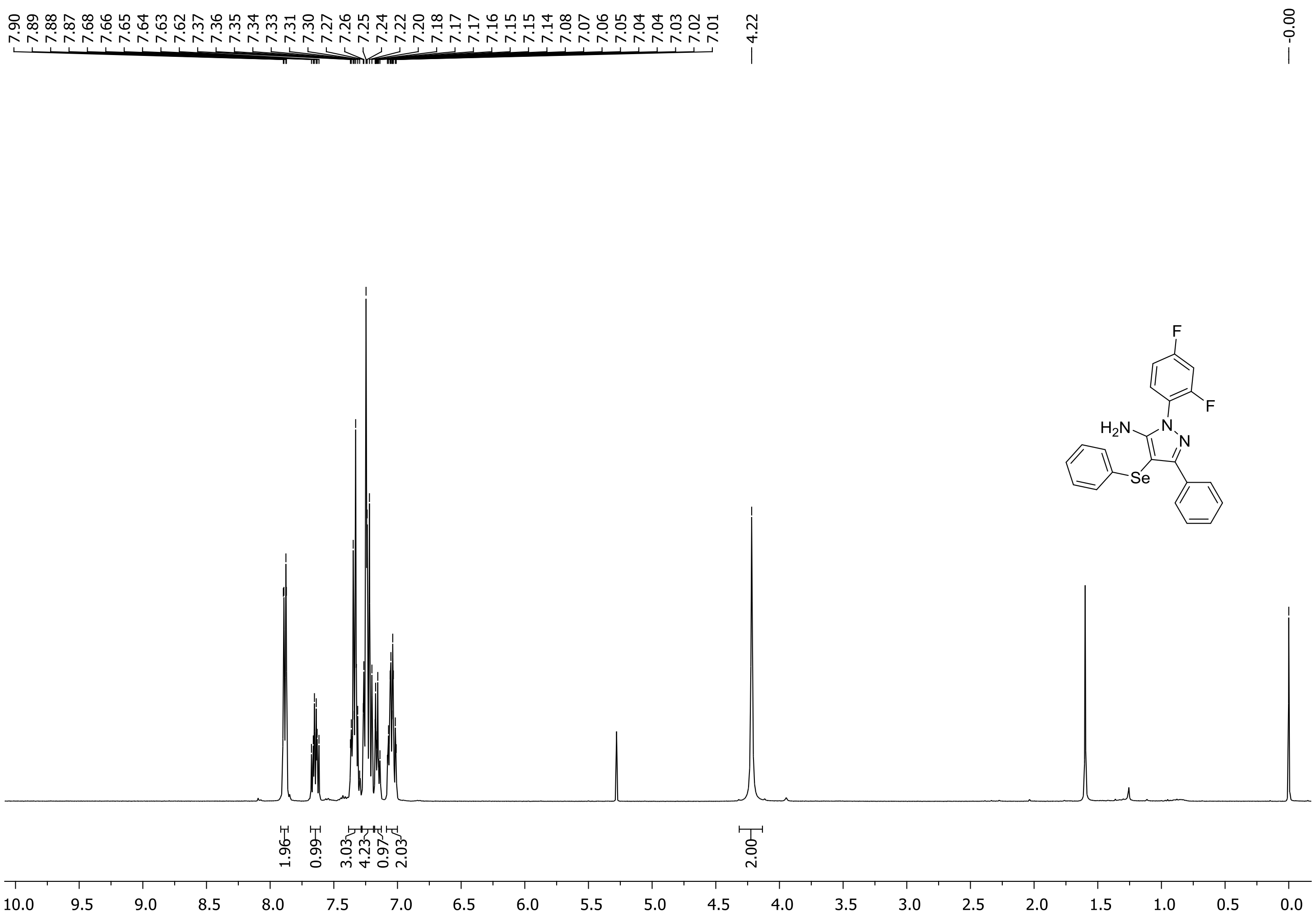

**Figure S17.** $^{1}$H NMR (400 MHz, $CDCl_3$) of the product **4j**.

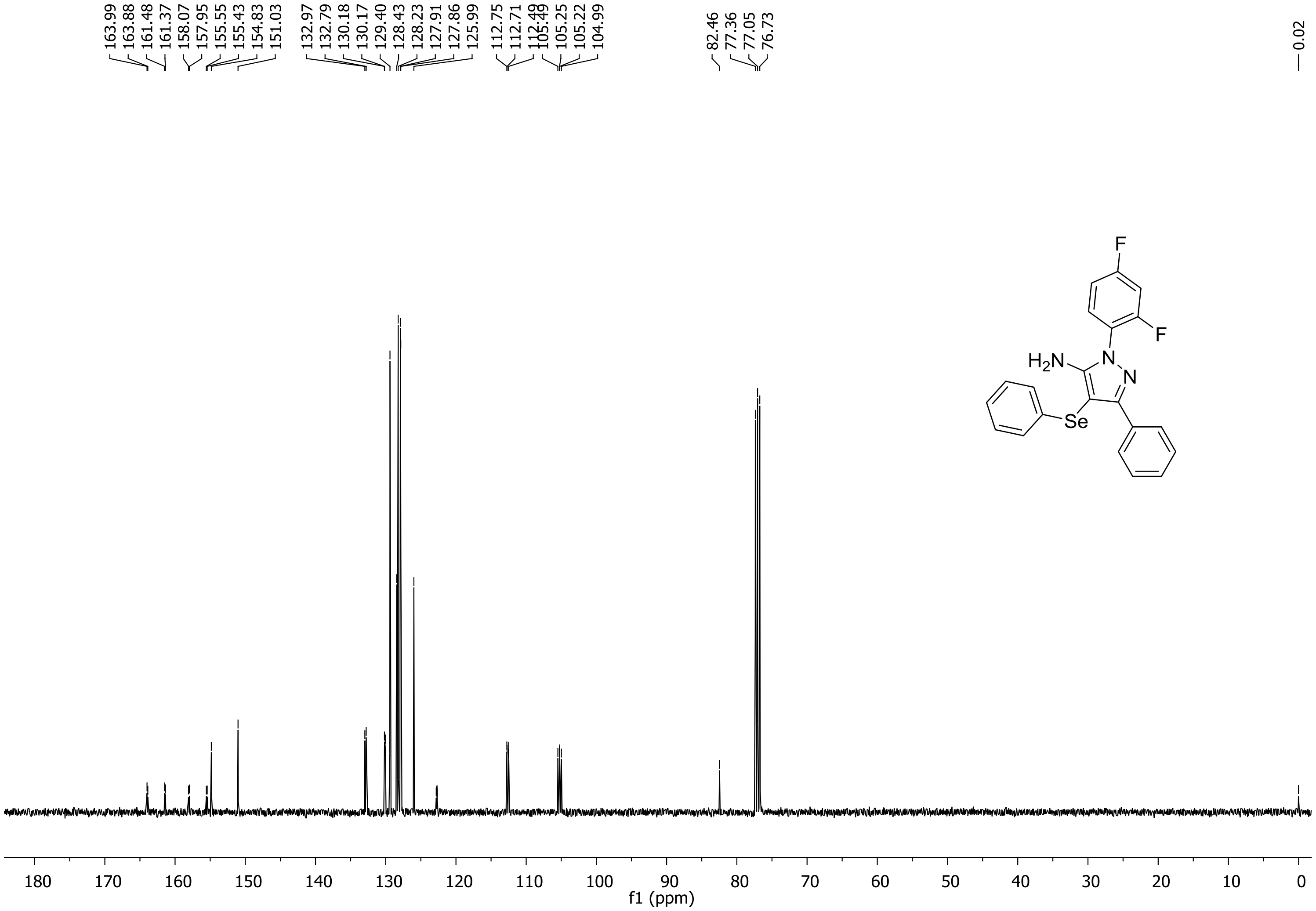


**Figure S18.** $^{13}$C NMR (100 MHz, $CDCl_3$) of the product **4j**.

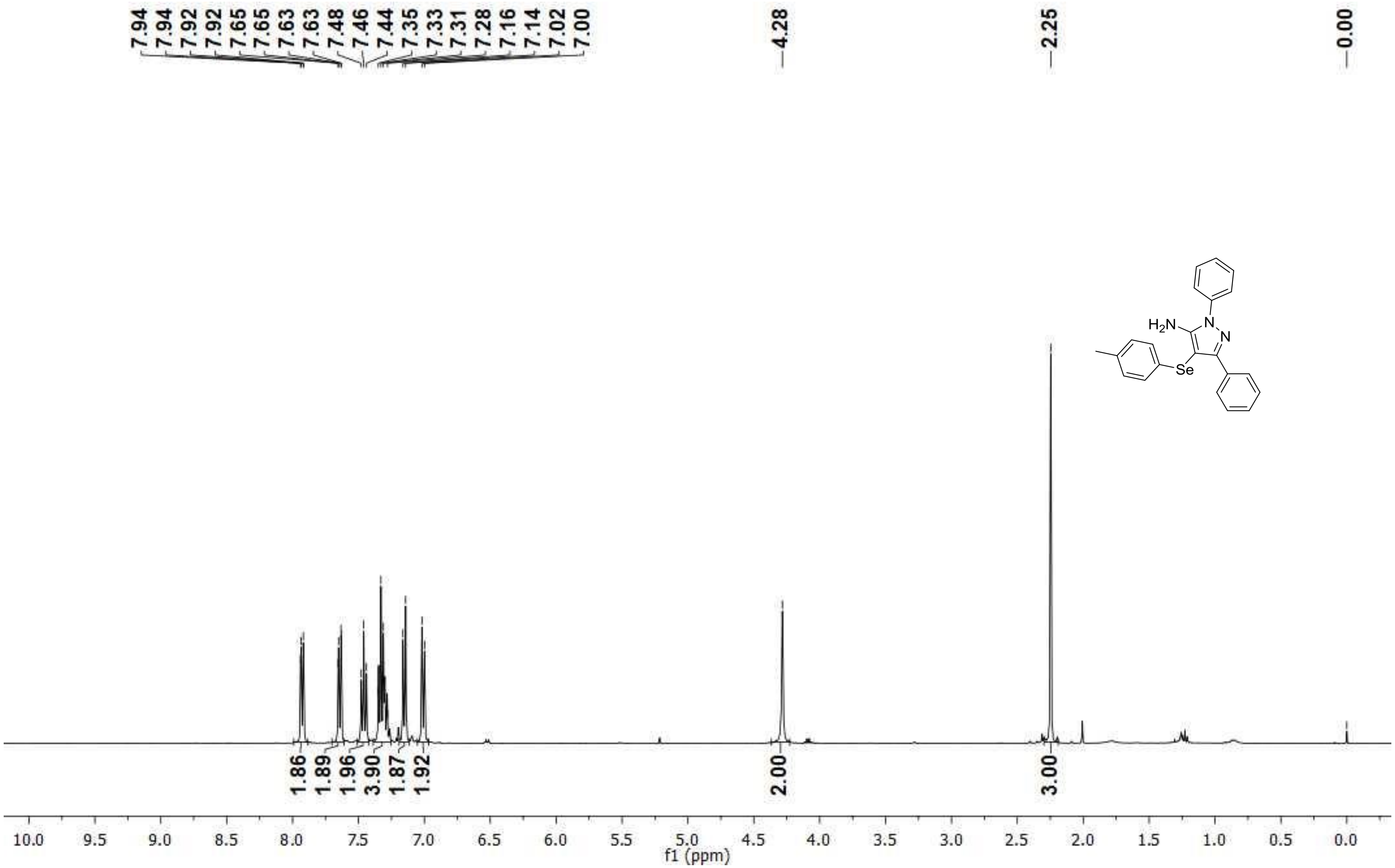


**Figure S19.** $^1$H NMR (400 MHz, $CDCl_3$) of the product **4l**.

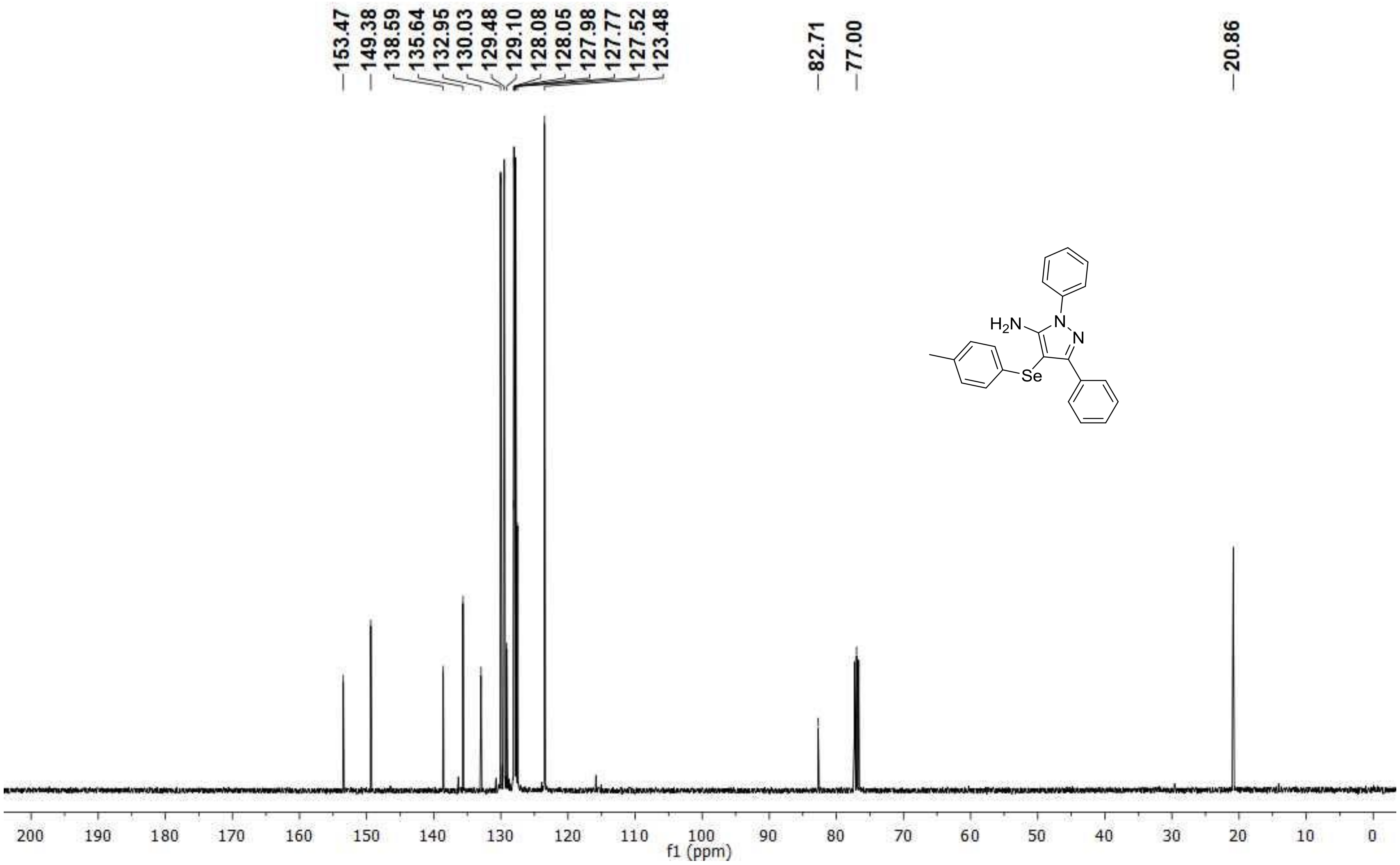


**Figure S20.** $^{13}$C NMR (100 MHz, $CDCl_3$) of the product **4l**.

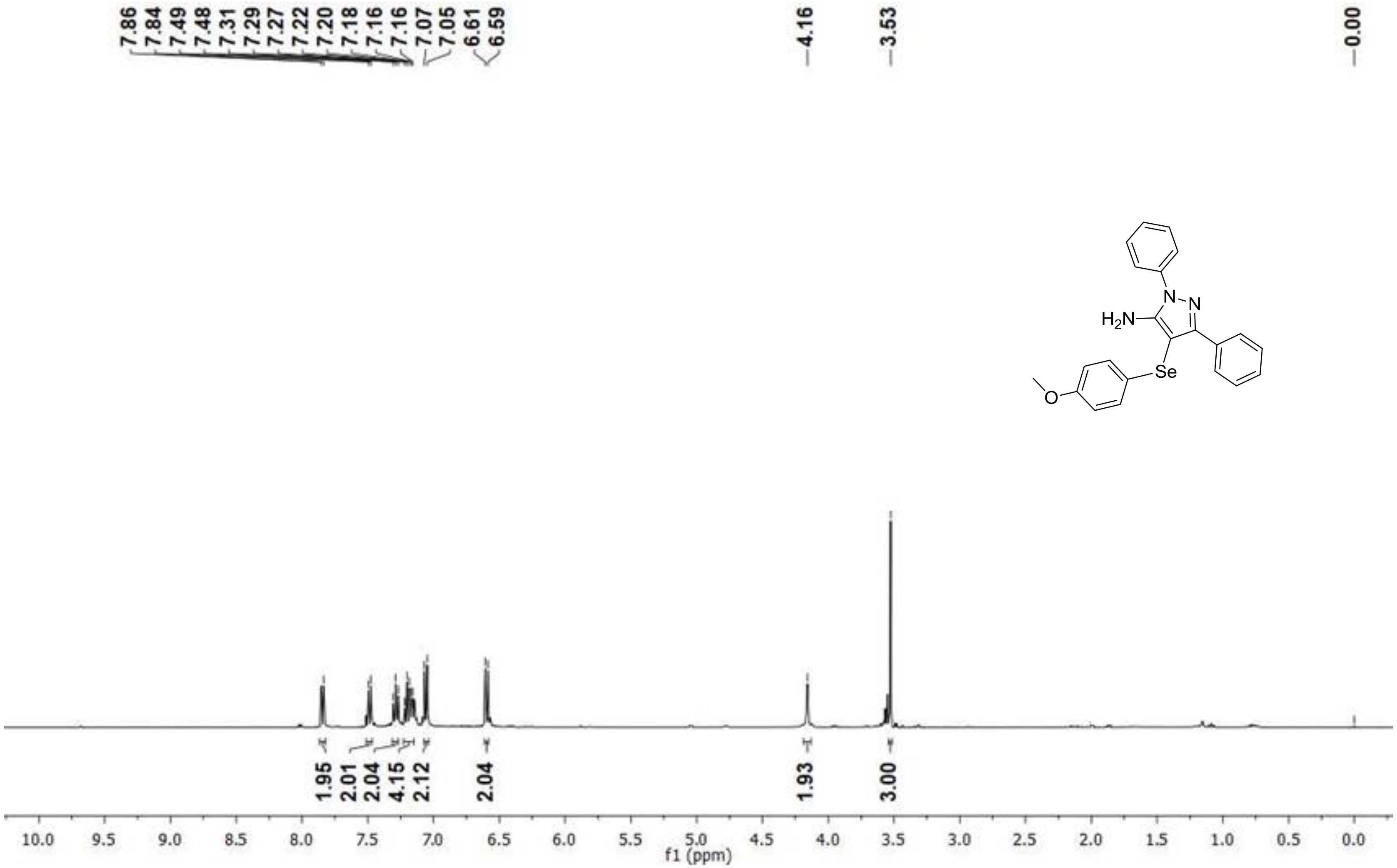


**Figure S21.** $^1$H NMR (400 MHz, $CDCl_3$) of the product **4m**.

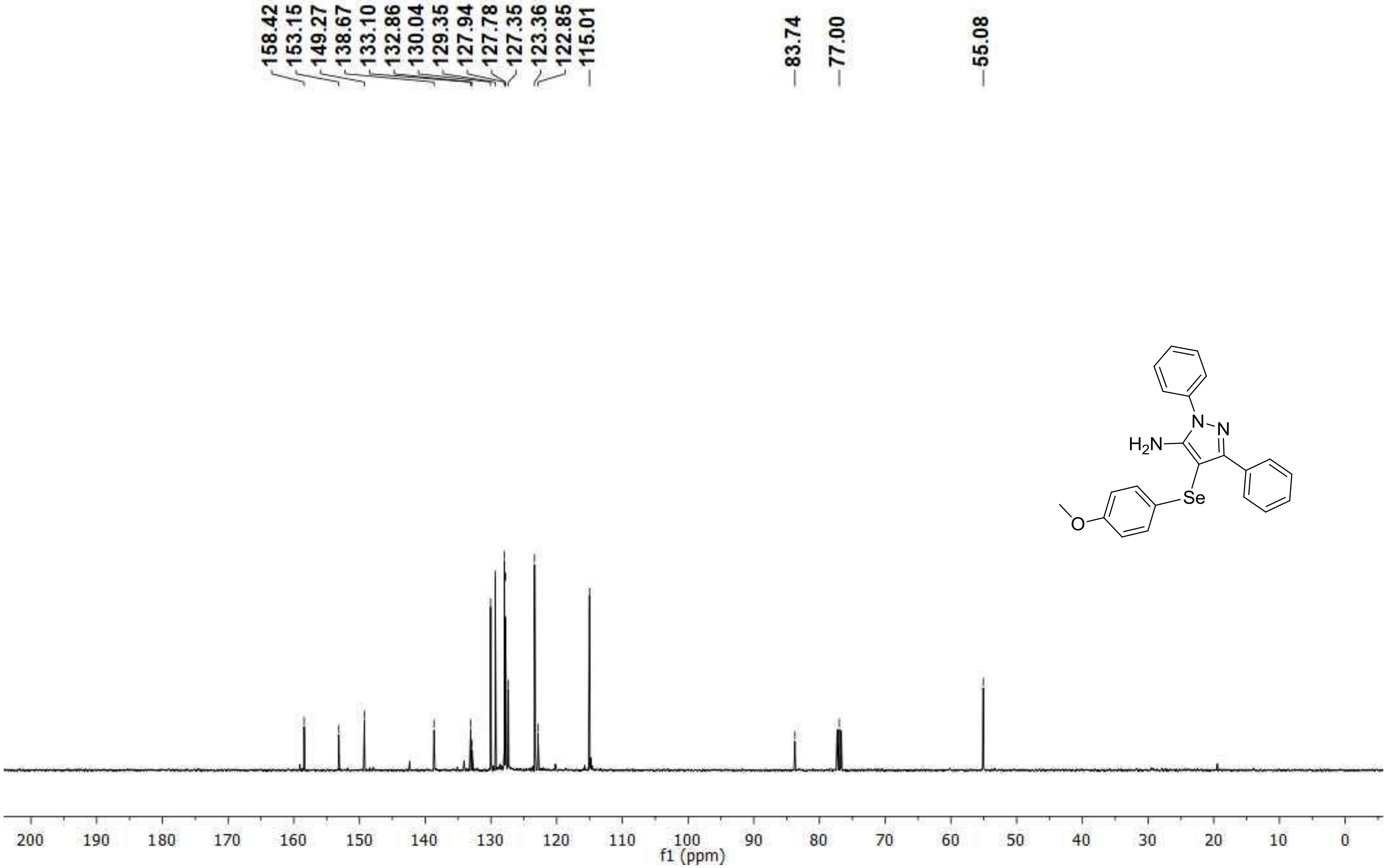


**Figure S22.** $^{13}$C NMR (100 MHz, $CDCl_3$) of the product **4m**.

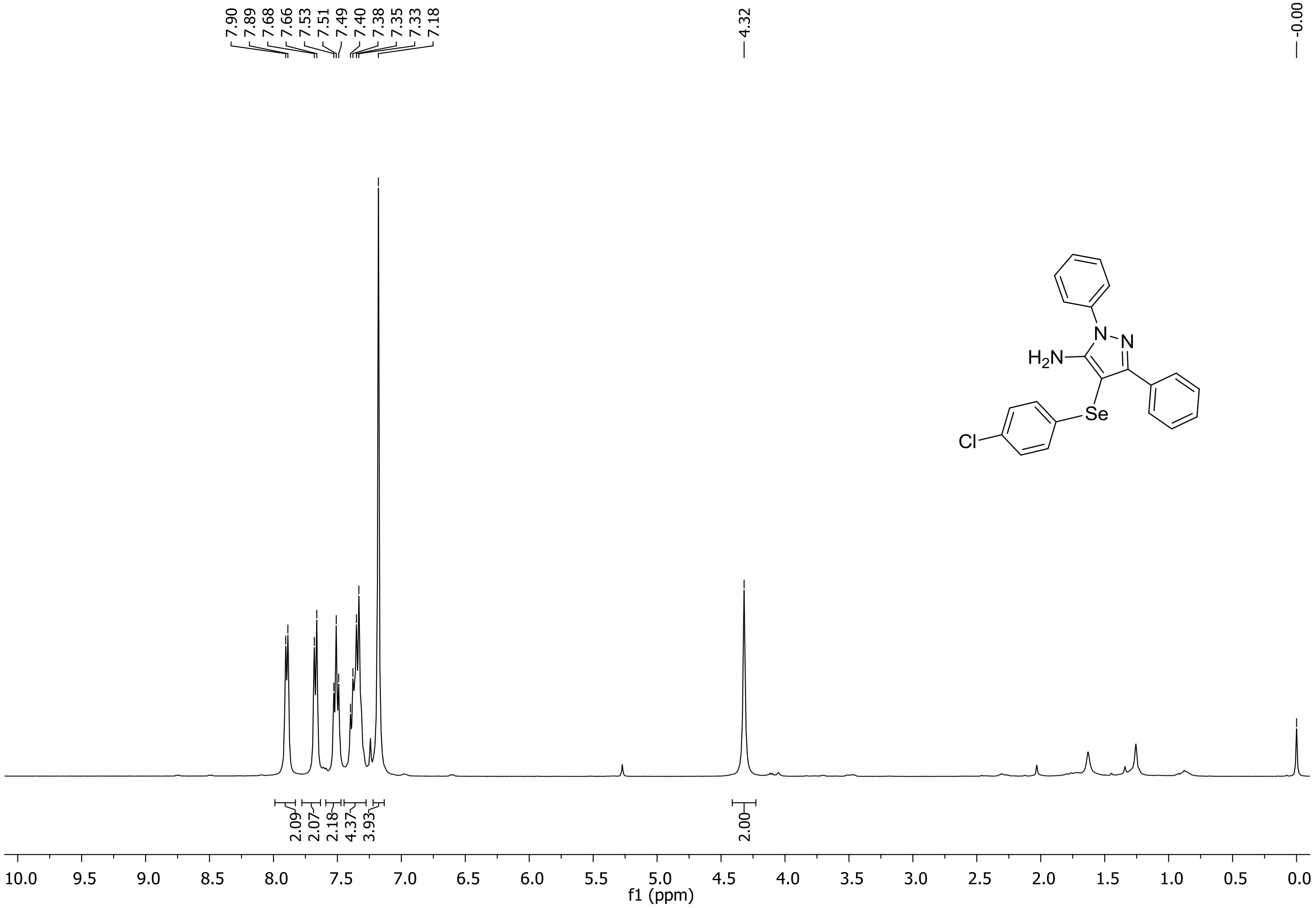


**Figure S23.** $^{1}H$ NMR (400 MHz, $CDCl_3$) of the product **4n**.

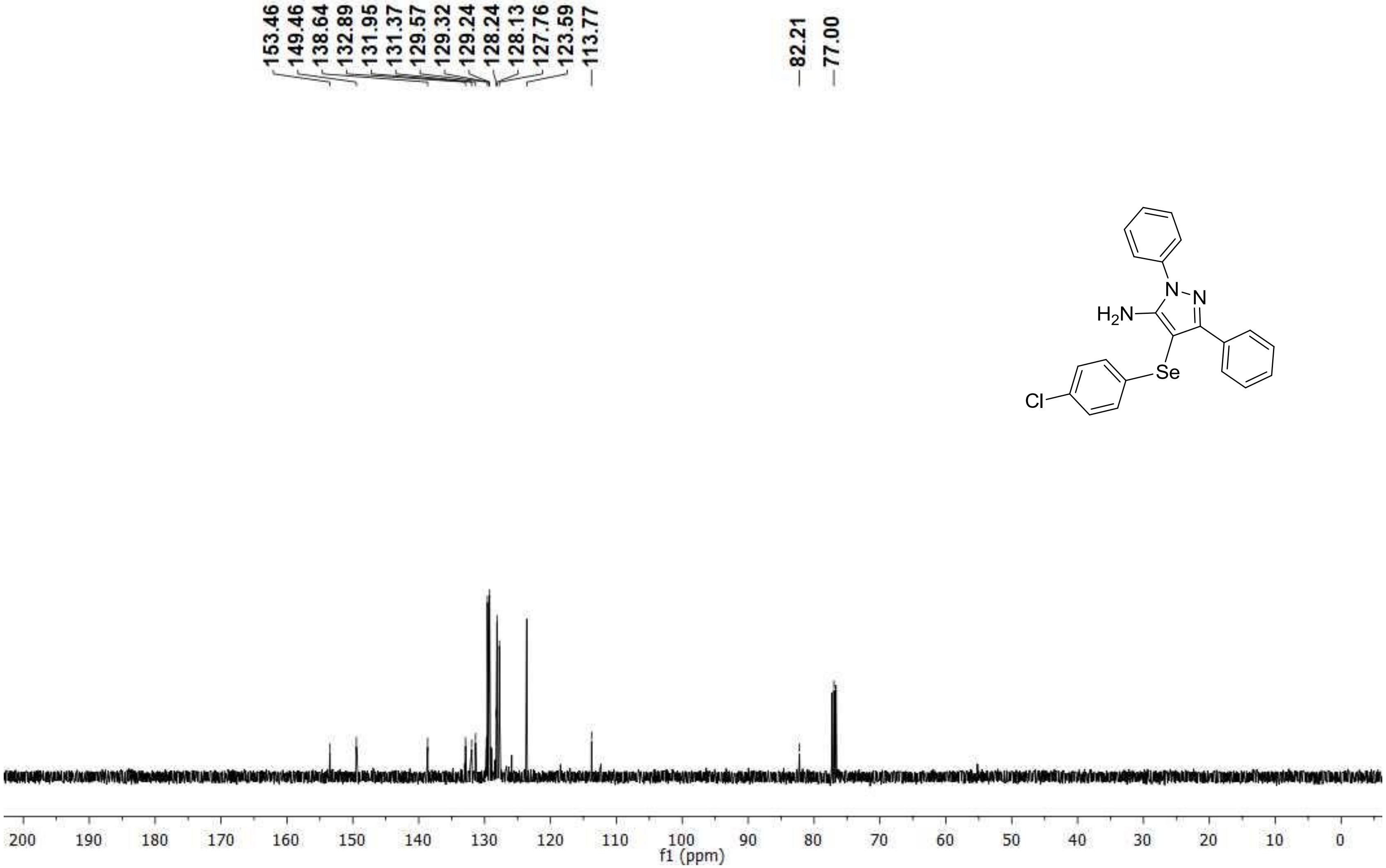


**Figure S24.** $^{13}C$ NMR (100 MHz, $CDCl_3$) of the product **4n**.

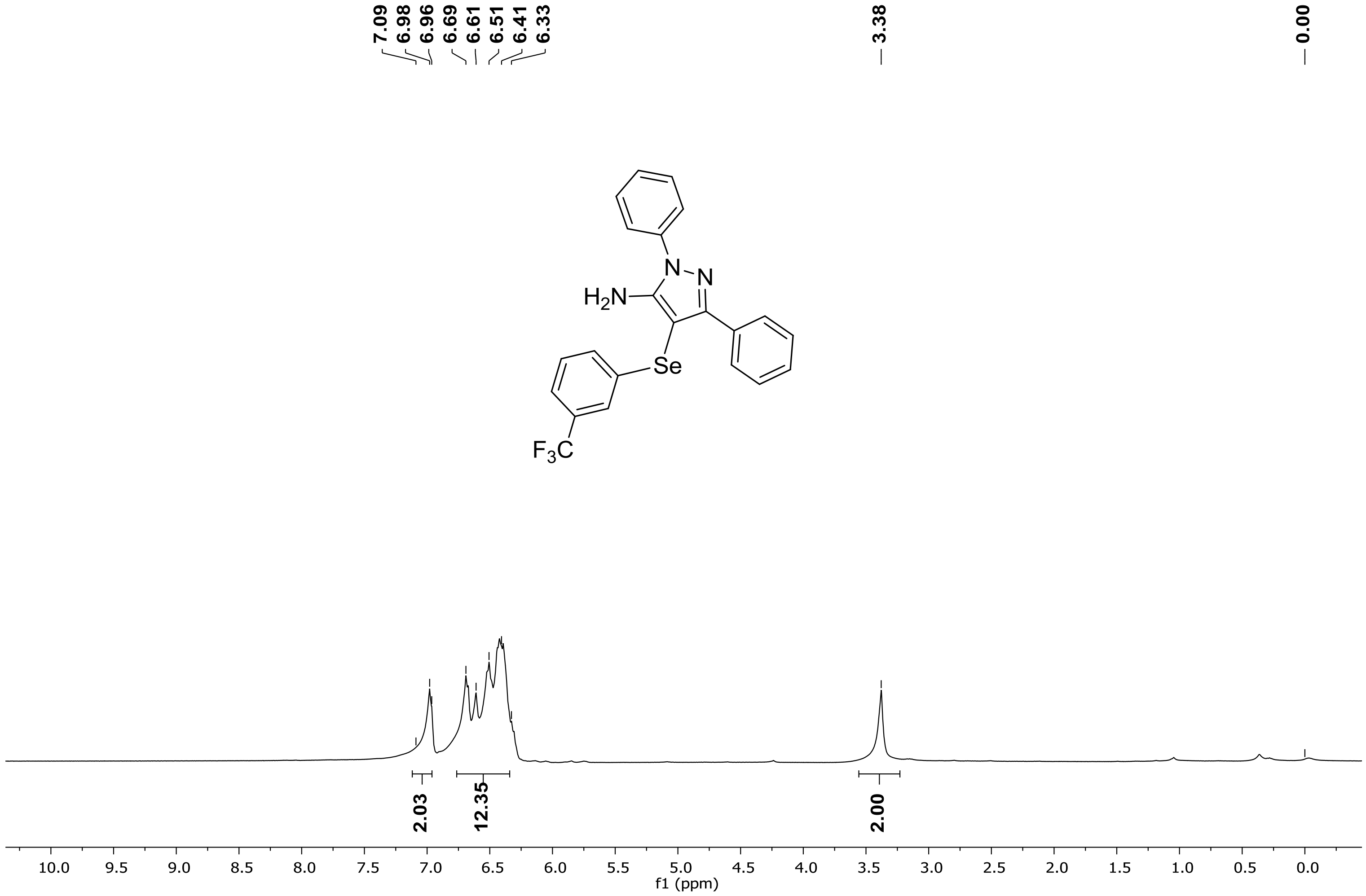


**Figure S25.** $^{1}$H NMR (400 MHz, $CDCl_3$) of the product **4o**

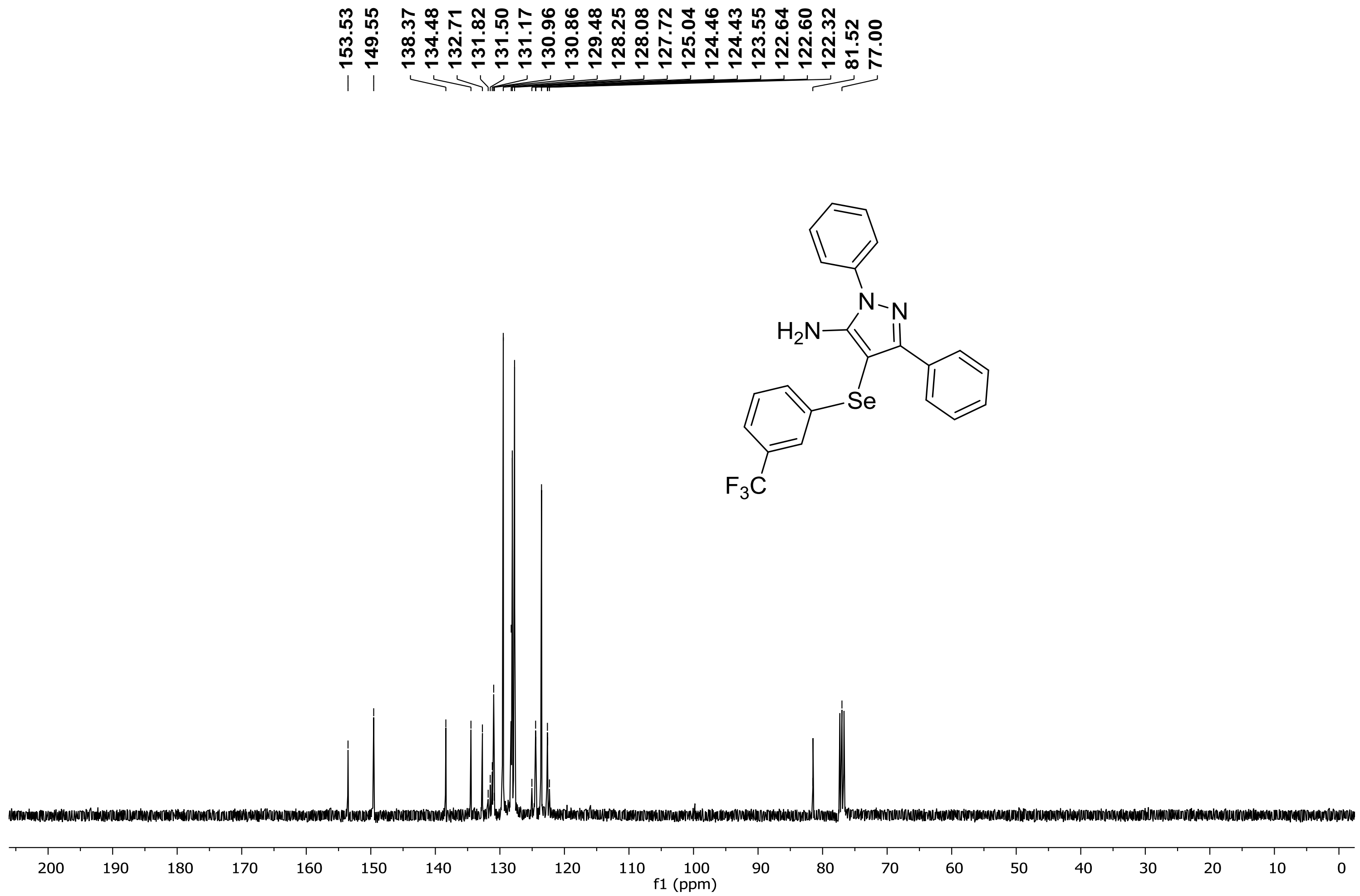


**Figure S26.** $^{13}$C NMR (100 MHz, $CDCl_3$) of the product **4o**.

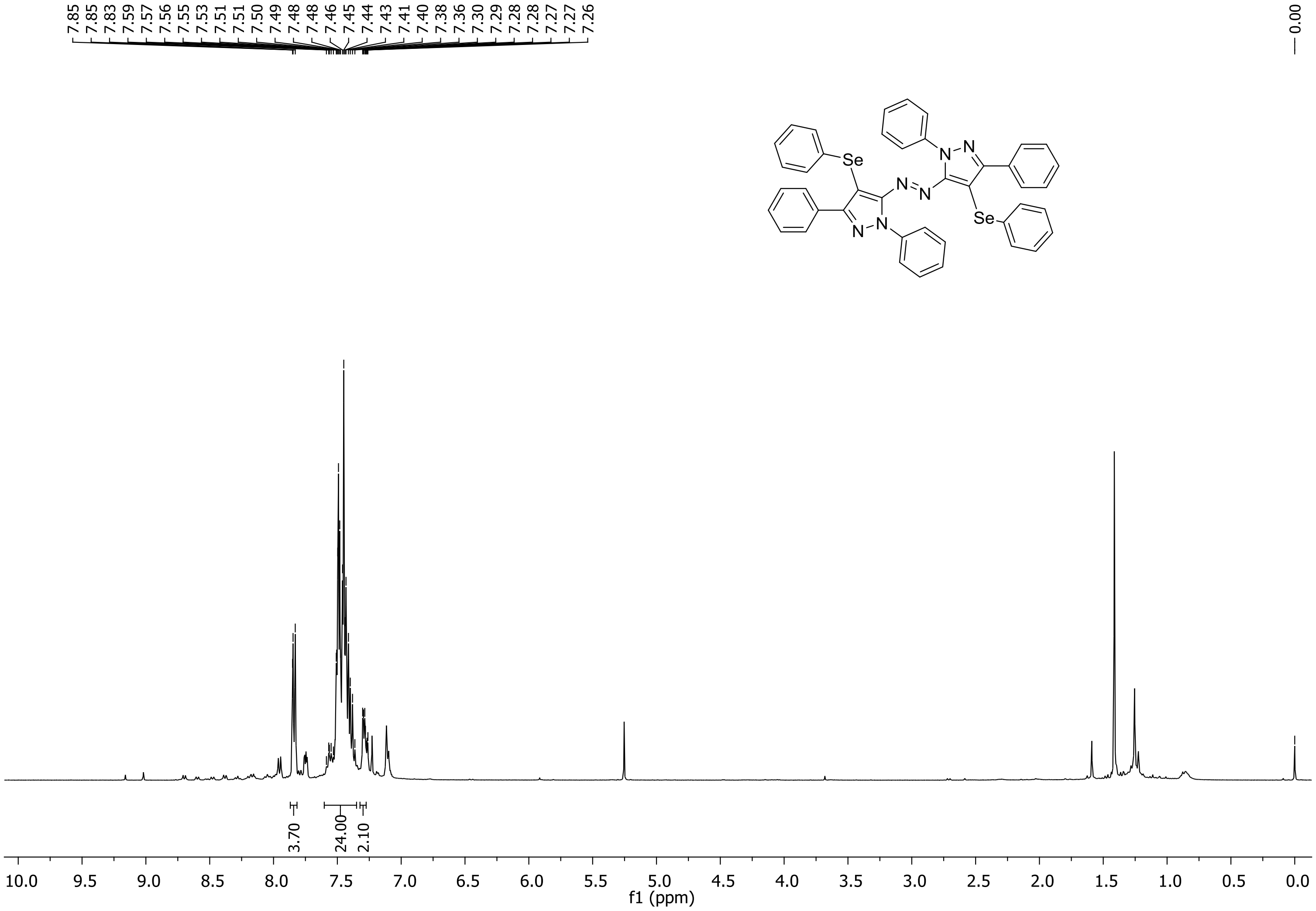


**Figure S27**. $^{1}$H NMR (400 MHz, $CDCl_3$) of the product **6**.

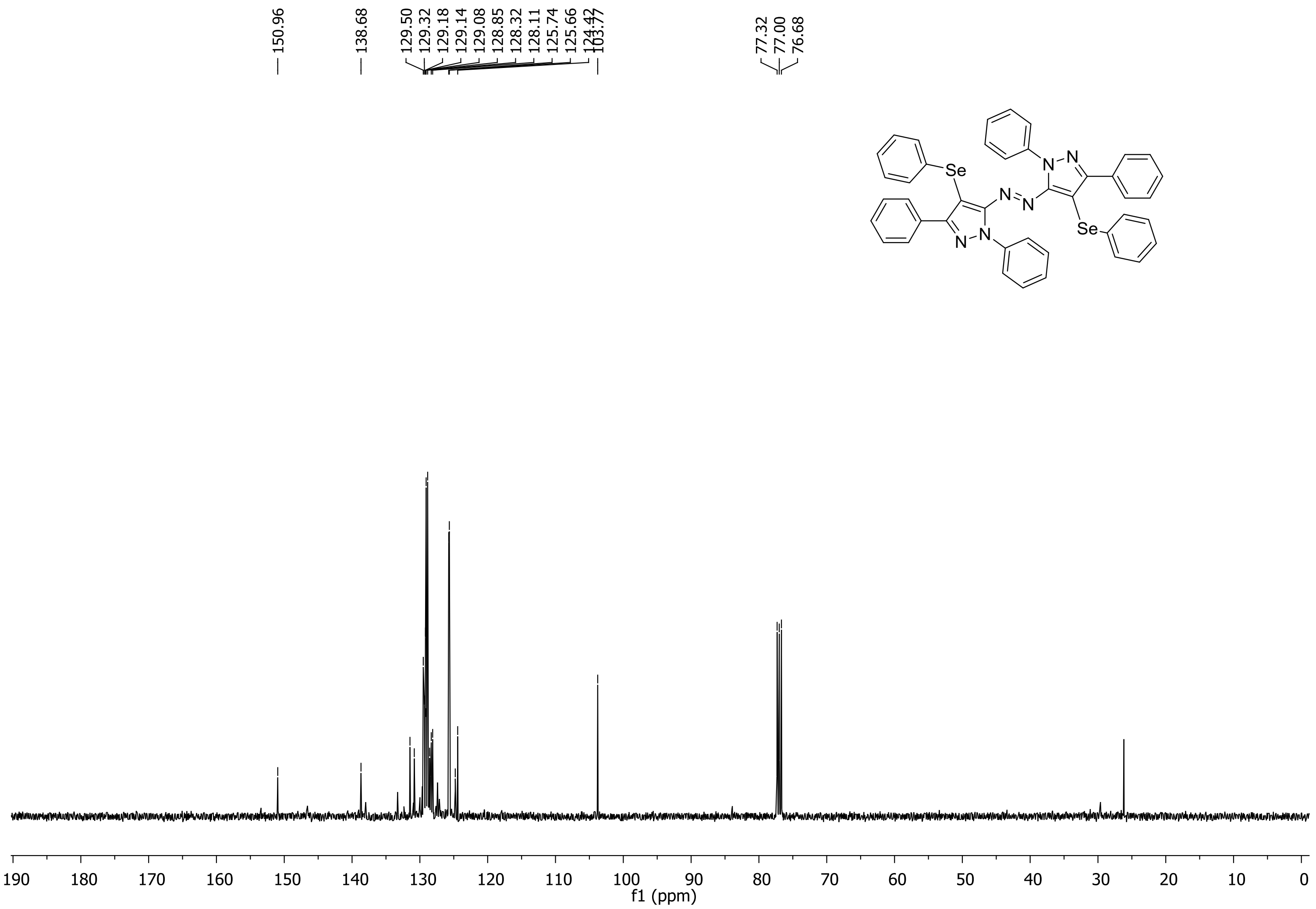


**Figure S28**. $^{13}$C NMR (100 MHz, $CDCl_3$) of the product **6**.
